# A high-throughput screen identifies that CDK7 activates glucose consumption in lung cancer cells

**DOI:** 10.1038/s41467-019-13334-8

**Published:** 2019-11-29

**Authors:** Chiara Ghezzi, Alicia Wong, Bao Ying Chen, Bernard Ribalet, Robert Damoiseaux, Peter M. Clark

**Affiliations:** 1 Crump Institute for Molecular Imaging, University of California, Los Angeles, CA 90095 USA; 2 Department of Molecular and Medical Pharmacology, University of California, Los Angeles, CA 90095 USA; 3 Department of Physiology, University of California, Los Angeles, CA 90095 USA; 4 California NanoSystems Institute, University of California, Los Angeles, CA 90095 USA; 5 Eli and Edythe Broad Center of Regenerative Medicine and Stem Cell Research, University of California, Los Angeles, CA 90095 USA

**Keywords:** Cancer metabolism, Non-small-cell lung cancer, Chemical genetics, Metabolic pathways, High-throughput screening

## Abstract

Elevated glucose consumption is fundamental to cancer, but selectively targeting this pathway is challenging. We develop a high-throughput assay for measuring glucose consumption and use it to screen non-small-cell lung cancer cell lines against bioactive small molecules. We identify Milciclib that blocks glucose consumption in H460 and H1975, but not in HCC827 or A549 cells, by decreasing SLC2A1 (GLUT1) mRNA and protein levels and by inhibiting glucose transport. Milciclib blocks glucose consumption by targeting cyclin-dependent kinase 7 (CDK7) similar to other CDK7 inhibitors including THZ1 and LDC4297. Enhanced PIK3CA signaling leads to CDK7 phosphorylation, which promotes RNA Polymerase II phosphorylation and transcription. Milciclib, THZ1, and LDC4297 lead to a reduction in RNA Polymerase II phosphorylation on the SLC2A1 promoter. These data indicate that our high-throughput assay can identify compounds that regulate glucose consumption and that CDK7 is a key regulator of glucose consumption in cells with an activated PI3K pathway.

## Abstract (web summary)

Many cancer cells have increased glucose consumption compared to normal cells, a feature that can be exploited therapeutically. Here, the authors carry out a chemical screen and identify compounds that selectively blocks glucose metabolism in non-small-cell lung cancer cell lines.

## Introduction

Elevated tumor glucose consumption, also known as the Warburg effect, is fundamental to most cancers^1–3^. The Warburg effect is induced by oncogenes or by the loss of tumor suppressors, and is exploited clinically to visualize cancer with ^18^F-2-fluoro-2-deoxyglucose (^18^F-FDG) PET^4–10^. Targeting glucose consumption in cancer alone or in combination with other therapies can limit cancer cell growth and induce apoptosis^11–16^. Glucose consumption in cancer is primarily targeted using 2-deoxyglucose (2-DG), a glucose analog that at high concentrations can function as a prodrug and can target the hexokinase enzymes that catabolize glucose^11–16^. However, 2-DG is poorly selective for cancer over non-transformed cells, leading to significant on-target toxicities in patients and limiting the use of 2-DG clinically^17^. Inhibitors that target the GLUT glucose transporters show promise but have yet to be tested in patients^18,19^.

Cancer cells, despite using the same biochemical enzymes as non-transformed cells to consume glucose, use different signaling pathways to regulate these enzymes^20–22^. Targeting these pathways may represent a strategy to block cancer cell glucose consumption while sparing healthy cells and may provide greater selectivity than directly targeting the enzymes that metabolize glucose. However, such an approach will require a deeper understanding of the pathways that regulate glucose consumption in cancer and new ways to target these pathways.

A major challenge of studying glucose consumption has been the lack of high-throughput methods for measuring this pathway. Standard assays use isotopically labeled glucose or glucose analogs (i.e. ^13^C-glucose, ^3^H-2-DG, ^18^F-FDG) or quantify changes in media glucose consumption and are low-throughput^21,23^. High-throughput assays, coupled to small molecule or genetic libraries, are a powerful method for identifying new regulators of a protein or pathway^24,25^.

Here, we adapted and optimized a luminescence-based glucose consumption assay into a high-throughput assay with the necessary properties for use in a high-throughput screen. We screened 3555 compounds against A549, H460, and HCC827 non-small-cell lung cancer (NSCLC) cells and identified 97 inhibitors of glucose consumption including the small molecule Milciclib. Milciclib inhibits glucose transport by blocking CDK7 from phosphorylating and activating RNA Polymerase II downstream of activated PIK3CA.

## Results

### A high-throughput assay for measuring glucose consumption

**Fig. 1:**
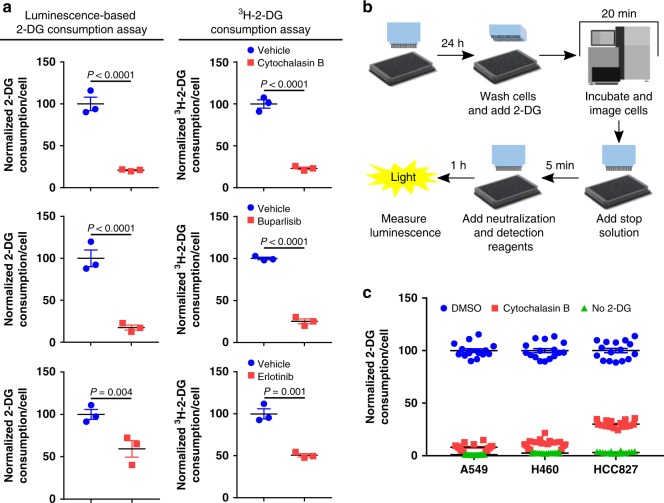
A high-throughput assay for measuring glucose consumption. **a** Glucose consumption in H460 (top and middle) and HCC827 (bottom) cells treated with Cytochalasin B (10 μM), Buparlisib (1 μM), Erlotinib (200 nM), or vehicle, measured by a luminescence-based 2-DG or a ^3^H-2-DG consumption assay. *n* = 3. *P* values determined by a two-way ANOVA test. **b** Schematic workflow of a luminescence-based high-throughput assay for measuring glucose consumption. **c** Glucose consumption, measured by a high-throughput assay, in A549, H460, and HCC827 cells treated with DMSO, Cytochalasin B (10 μM), or without 2-DG. *n* = 16. Data are plotted as mean ± SEM.

Glucose consumption is usually evaluated by measuring the accumulation of long-lived radio-labeled or stable isotope-labeled glucose analogs into cells^21,23^, methods that would be challenging to implement in a high-throughput assay. Luminescence is a preferred readout for high-throughput screens, and a recent study described a luminescence-based assay for measuring cellular glucose consumption^26,27^. This assay measures 2-DG-6-phosphate levels using a coupled enzyme assay in cells treated with 2-DG^27^ (Supplementary Fig. 1). We tested the sensitivity of this assay to drug-induced changes in glucose consumption and compared these results to results obtained using ^3^H-2-DG. Cytochalasin B is a small molecule inhibitor of the GLUT glucose transporters^28^. The luminescence-based glucose consumption assay measured a 79.1 ± 0.7% (mean ± standard error of the mean (SEM)) decrease in glucose consumption in Cytochalasin B-treated cells, which was similar to the 76.8 ± 1.5% decrease in glucose consumption measured using ^3^H-2-DG (Fig. 1a), consistent with previous studies^27^. Both PI3K and EGFR activate glucose consumption in cancer^9,22^. Using the luminescence-based glucose consumption assay, we identified that the PI3K inhibitor Buparlisib and the EGFR inhibitor Erlotinib blocked glucose consumption by 82.4 ± 3.0% and 40.7 ± 9.7%, respectively. Similar results were obtained using ^3^H-2-DG (a 74.9 ± 3.0% and 49.3 ± 2.0% decrease with Buparlisib and Erlotinib, respectively; Fig. 1a). These data indicate that the luminescence-based glucose consumption assay can sensitively and accurately measure drug-induced changes in glucose consumption.

Converting this low-throughput, luminescence-based glucose consumption assay into a miniaturized, high-throughput assay required a number of significant modifications. Potential liabilities included wash steps to remove endogenous glucose, which could lead to variable cell loss, and consecutive reagent addition steps, which could compound liquid handling errors. Simply applying this assay as described in the literature^27^ to an automated, high-throughput workflow with volumes scaled proportionate to the surface area of wells in a 384-well plate produced an assay with a *Z*-factor of −1.59 ± 0.29 with Cytochalasin B as the positive control. However, multiple rounds of optimization yielded a robust protocol: cells expressing a nuclear-localized blue-fluorescent protein (BFP^+^ cells) are plated into wells of a 384-well plate containing small molecules or DMSO in media. Following a 24 h incubation, the cells are quickly washed with 1x PBS containing 0.25% BSA, and 2-DG is added. Over a 20 min period in which the cells are incubated with the 2-DG, an automated fluorescent microscope images and counts the BFP^+^ cells in each well. A stop buffer is added followed by a neutralization and detection reagent, and the luminescence is measured on a plate reader (Fig. 1b). Results are reported as luminescence per BFP^+^ cell. Key features of this protocol include the fluorescent imaging step, which removed any variability in the luminescence output caused by differential cell loss during the washes; the addition of BSA to the wash solutions to decrease surface tension, which increased assay reproducibility; and an optimized ratio of stop buffer to neutralization and detection reagent, which maximized output while minimizing background signal. Across three-cell lines, the assay yielded a *Z*-factor of 0.75 ± 0.02 and 0.59 ± 0.05 using no 2-DG and Cytochalasin B as the positive controls, respectively (Fig. 1c). These results suggest that our high-throughput assay has the necessary properties for incorporation into a high-throughput screen.

### Discovering inhibitors of glucose consumption in NSCLC cells

**Fig. 2:**
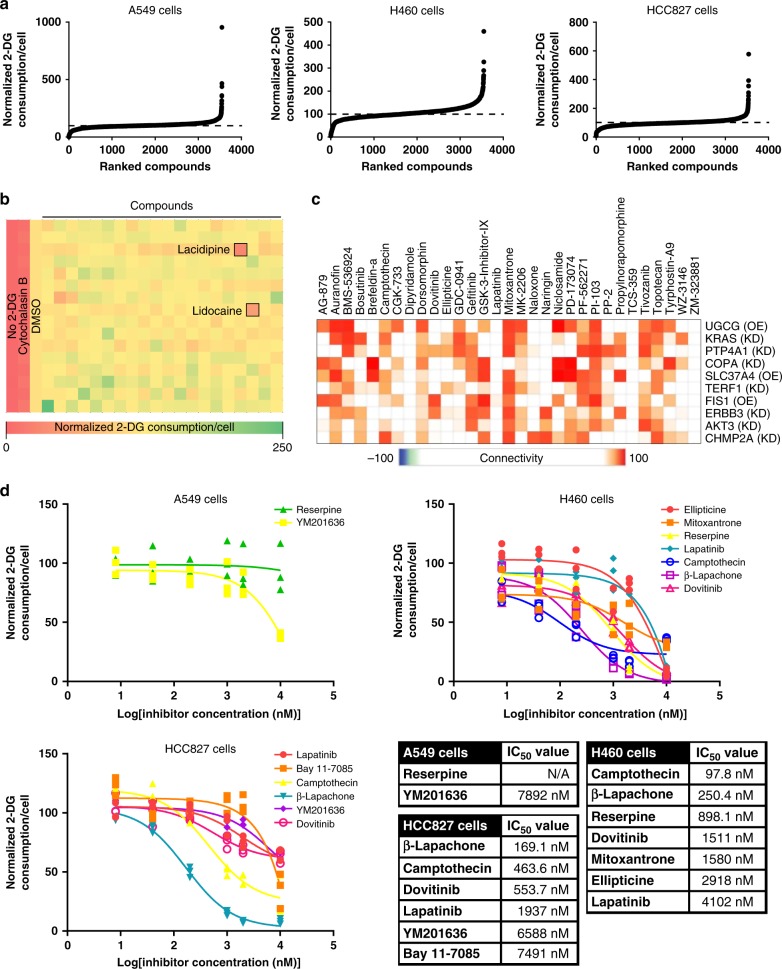
A high-throughput screen identifies inhibitors of NSCLC glucose consumption. **a** Waterfall plots of glucose consumption in A549, H460, and HCC827 cells treated with one of 3555 small molecule inhibitors. *n* = 1. **b** Example heat map of glucose consumption from a 384-well plate of H460 cells treated with small molecules from the NIH clinical collection. **c** Genetic alterations connected to the small molecules we identified as inhibitors of glucose consumption. Analysis performed using the Connectivity Map. OE overexpression, KD knockdown. **d** Glucose consumption dose response curves and values for a subset of the compounds identified in the high-throughput screen. Reserpine in A549 cells is used as a negative control. *n* = 3 per concentration except *n* = 2 for H460 cells treated with Reserpine.

NSCLC cells have elevated glucose consumption, and inhibiting glucose consumption in these cells limits their growth and sensitizes them to chemotherapy^11,13,29^. We screened A549, H460, and HCC827 NSCLC cells against 3555 bioactive small molecules from four small molecule libraries: the Selleck Chemicals kinase inhibitor library, the Prestwick FDA-approved drug library, the LOPAC collection, and the NIH clinical collection. A549 cells carry a G12S KRAS mutation, H460 cells carry a Q61H KRAS and E545K PIK3CA mutations, and HCC827 cells carry an EGFR Exon 19 deletion^30^. This screen yielded a list of small molecules and their effects on glucose consumption in each cell line (Fig. 2a: waterfall plots; Fig. 2b: example heat map). While most of the compounds had little effect on glucose consumption, a small number of compounds altered cellular glucose consumption. Our high-throughput assay has a standard deviation of 7.6% (Fig. 1c), so we chose a 50% decrease in glucose consumption (e.g. >6 standard deviations from the mean) as our cut-off for evaluating potential hits. Using this cut-off, we identified 34, 95, and 66 putative inhibitors of glucose consumption in A549, H460, and HCC827 cells, respectively. 8, 61, and 44 of these putative inhibitors were validated in A549, H460, and HCC827 cells, respectively, following a secondary screen. This yielded a reconfirmation rate of 58% and provided a list of validated inhibitors of glucose consumption in these cell lines (Supplementary Table 1). Only 16 of these small molecules overlap across two cell lines, potentially suggesting that these genetically distinct cell lines use different mechanisms to promote glucose consumption.

Our list of validated hits includes known and novel inhibitors of glucose consumption including kinase inhibitors (Lapatinib, Dovitinib, and Gefitinib), topoisomerase inhibitors (Ellipticine and (S)-(+)-camptothecin), and dopamine receptor agonists (R-(−)-propylnorapomorphine and l-apomorphine) (Supplementary Table 1). Approximately 30% of the validated hits are profiled in the Connectivity Map^31^. Consistent with their shared ability to limit glucose consumption, the inhibitors we identified link to overexpression of UGCG and SLC37A4, two genes whose protein products consume or sequester metabolites of glucose (Fig. 2c). Additionally, knockdown or inhibition of various proteins involved in receptor tyrosine kinase signaling are connected to the inhibitors we identified (Fig. 2c, Supplementary Fig. 2), consistent with a role for receptor tyrosine kinases in driving glucose consumption in cancer^21,22^. We cannot readily explain other connected genetic alterations including knockdown of COPA, TERF1, and CHMP2A, and overexpression of FIS1, but these insights could form the basis for future studies. The only overlap between these results and connections identified from a control set of compounds was ERBB3 knockdown.

We further analyzed 14 of our validated hits by determining glucose consumption IC_50_ values. Based on the results of our high-throughput screen, Reserpine does not affect glucose consumption in A549 cells, so we used it as a negative control. IC_50_ values could be determined for all of the compounds except for our negative control (Fig. 2d). In these targeted analyses and in the high-throughput screens, there was no consistent connection between whether, 24 h post-drug treatment, a small molecule inhibitor blocked glucose consumption and whether that same inhibitor blocked cell growth although specific examples of this connection do exist (Supplementary Fig. 3). Collectively, these data indicate that our high-throughput assay can be used to identify small molecule inhibitors of glucose consumption. The results of this screen are a list of known and novel inhibitors of glucose consumption in NSCLC (Supplementary Table 1).

### Milciclib inhibits glucose consumption in H460 cells

**Fig. 3:**
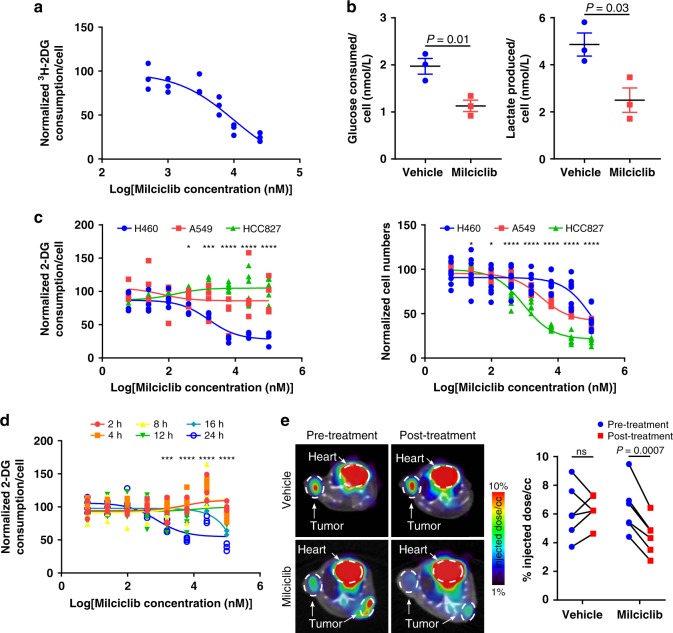
Milciclib inhibits glucose consumption in H460 NSCLC cells. **a** Glucose consumption dose response curve measured by ^3^H-2-DG consumption in H460 cells treated with Milciclib. *n* = 3. **b** Glucose consumption and lactate production measured from cell media by a bioprofile basic analyzer in H460 cells treated with vehicle or Milciclib (10 μM). *n* = 3. *P* values determined by unpaired *t* tests. **c** Glucose consumption (left) and cell growth (right) in H460, A549, and HCC827 cells treated with Milciclib. Glucose consumption: H460 and A549, *n* = 4; HCC827, *n* = 6. Cell growth: H460 and A549, *n* = 8; HCC827, *n* = 6. *P* values determined by a two-way ANOVA test. **d** Glucose consumption in H460 cells at different time points post-Milciclib treatment. *n* = 4. *P* values determined by a two-way ANOVA test. **e** ^18^F-FDG PET images (left) and quantification (right) of H460 cell xenografts in mice pre-treatment and post-treatment with vehicle or Milciclib (30 mg kg^-1^). *n* = 6. *P* values determined by paired *t* tests. ns: not significant. *P < 0.05; ***P < 0.001; ****P < 0.0001. Data are plotted as mean ± SEM.

Milciclib is an inhibitor of CDK2, CDK4, CDK7, and TRKA^32,33^ and in our high-throughput screen we found that it blocks glucose consumption specifically in H460 cells. This suggests a potential mechanistic connection between cell cycle regulation and metabolism in NSCLC through a cyclin-dependent kinase and that Milciclib may block glucose consumption downstream of a specific genetic alteration and thus show specificity for some cancers over healthy cells. We further evaluated whether Milciclib blocked H460 cell glucose consumption in cell culture and in vivo using additional and orthogonal assays. Consistent with the results of our high-throughput assay, 10 µM Milciclib decreased glucose consumption in H460 cells by 65.8 ± 3.1% and 42.7 ± 6.2% as evaluated by measuring ^3^H-2-DG consumption or media glucose levels, respectively (Fig. 3a, b). Cancer cells catabolize glucose to lactate^8^. Milciclib decreased H460 lactate production by 48.7 ± 10.6% (Fig. 3b). Milciclib blocked glucose consumption in H460 but not in A549 or HCC827 cells even though Milciclib-inhibited cell growth in all three cell lines (Fig. 3c). Milciclib blocked glucose consumption in H460 cells after 16 h but before 24 h of treatment (Fig. 3d). In vivo, Milciclib blocked glucose consumption in H460 cell xenografts by 30.4 ± 2.5%, as assessed using ^18^F-FDG PET imaging, without affecting glucose consumption in the mouse brain, heart, liver, or muscle (Fig. 3e, Supplementary Fig. 4). This suggests that Milciclib may inhibit glucose consumption selectively in cancer. Vehicle treatment did not significantly affect H460 cell xenograft glucose consumption (6.0 ± 6.0% increase) (Fig. 3e). Collectively, these data provide strong evidence that Milciclib inhibits glucose consumption selectively in H460 cells in cell culture and in vivo.

### Milciclib blocks GLUT expression and glucose transport

**Fig. 4:**
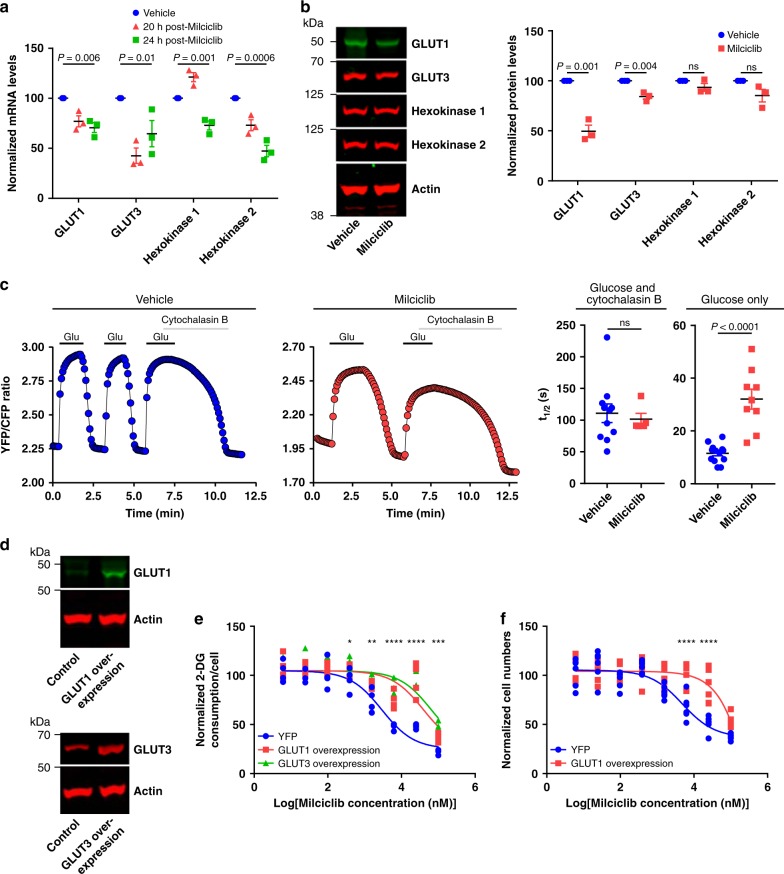
Milciclib blocks glucose consumption by limiting glucose transport. **a** mRNA levels from H460 cells treated with vehicle or Milciclib (10 μM). *n* = 3. *P* values determined by one-way ANOVA tests. **b** Immunoblots (left) and quantification (right) of lysate from H460 cells treated with vehicle or Milciclib (10 μM). *n* = 3. *P* values determined by unpaired *t* tests. **c** Representative FRET traces (left and middle) and quantification (right) of H460 cells treated with vehicle or Milciclib (10 μM). Glu: glucose. Glucose and Cytochalasin B: Vehicle, *n* = 11; Milciclib, *n* = 5. Glucose only: Vehicle, *n* = 13; Milciclib, *n* = 9. *P* values determined by unpaired *t* tests. **d** GLUT1 and GLUT3 protein levels in H460 cells transfected with a control (YFP) or a GLUT1 or GLUT3 overexpression plasmid. *n* = 2. **e** Glucose consumption dose response curves in H460 cells that overexpress YFP, GLUT1, or GLUT3 and that were treated with Milciclib. *n* = 4. *P* values determined by a two-way ANOVA test. **f** Cell growth dose response curves in H460 cells that overexpress YFP or GLUT1 and that were treated with Milciclib for 48 h. *n* = 6. *P* values determined by a two-way ANOVA test. ns: not significant. *P < 0.05; **P < 0.01; ***P < 0.001; ****P < 0.0001. Data are plotted as mean ± SEM.

Cancer cells predominantly express SLC2A1 (GLUT1) and SLC2A3 (GLUT3) to transport glucose across the cell membrane and Hexokinase 1 and Hexokinase 2 to phosphorylate glucose^34,35^. GLUT1, GLUT3, and Hexokinase 2 mRNA levels were lower in H460 cells treated with Milciclib compared to vehicle-treated cells (GLUT1, GLUT3, and Hexokinase 2 mRNA levels after 20 h: 76.9 ± 5.5%, 42.5 ± 7.7%, and 67.8 ± 3.4% of control values, respectively; GLUT1, GLUT3, and Hexokinase 2 mRNA levels after 24 h: 70.6 ± 4.9%, 64.5 ± 13.1%, and 47.2 ± 5.8% of control values, respectively; Fig. 4a). Hexokinase 1 mRNA levels were higher at 20 h and lower at 24 h post-Milciclib treatment compared to vehicle-treated cells (20 h: 121.1 ± 4.5% of control values; 24 h: 71.1 ± 7.1% of control values; Fig. 4a). Milciclib decreased GLUT1, GLUT3, Hexokinase 1, and Hexokinase 2 protein levels in H460 cells to 49.6 ± 6.1%, 84.3 ± 2.6%, 93.5 ± 3.9%, and 85.3 ± 6.3% of control values, respectively (Fig. 4b**;** Supplementary Fig. 5). In A549 and HCC827 cells, Milciclib had no significant effect on or slightly increased GLUT1, GLUT3, Hexokinase 1, or Hexokinase 2 protein levels (Supplementary Fig. 6). We further analyzed the effect of Milciclib on glucose transport and hexokinase activity in H460 cells using a FRET-based glucose reporter^36,37^. The rate of change in the FRET ratio following removal of glucose alone measures the combined activity of the glucose transporters and the hexokinases^38^. The rate of change in the FRET ratio following removal of glucose in the presence of Cytochalasin B measures exclusively the hexokinase activity. Milciclib did not significantly affect the rate of change of the FRET ratio following removal of glucose in the presence of Cytochalasin B (*t*_1/2_ = 110.9 ± 14.6 and 101.5 ± 9.2 s for vehicle and Milciclib, respectively; Fig. 4c), suggesting that Milciclib does not affect hexokinase activity. However, Milciclib significantly decreased the rate of change of the FRET ratio following removal of glucose without Cytochalasin B treatment (*t*_1/2_ = 11.5 ± 1.0 and 32.1 ± 3.8 s for vehicle and Milciclib, respectively; Fig. 4c), suggesting that Milciclib limits glucose consumption by decreasing the rate of glucose transport.

If Milciclib blocks glucose consumption by decreasing GLUT transporter levels, then overexpression of GLUT1 or GLUT3 should rescue this effect. H460 cells that overexpress GLUT1, GLUT3, or YFP were treated with vehicle or Milciclib, and glucose consumption was measured 24 h post-drug treatment. GLUT1 and GLUT3 overexpression increased GLUT1 and GLUT3 protein levels compared to YFP overexpression (Fig. 4d). GLUT1 and GLUT3 overexpression also shifted the Milciclib glucose consumption IC_50_ value by over 10-fold compared to cells overexpressing YFP without significantly affecting how much Milciclib alters cell growth 24 h post-Milciclib treatment (normalized cell numbers of YFP, GLUT1, and GLUT3 overexpression cells treated with Milciclib: 69.4 ± 3.4, 62.8 ± 3.9, and 60.7 ± 3.8, respectively; Fig. 4e). Additionally, GLUT1 overexpression decreased the ability of Milciclib to inhibit cell growth at 48 h post-drug treatment (Fig. 4f). This may suggest that part of the mechanism through which Milciclib blocks cell growth is by decreasing glucose consumption although additional experiments will be required to fully evaluate this possibility. Collectively our data suggest that Milciclib decreases glucose consumption in H460 cells by downregulating GLUT1 and GLUT3 mRNA and protein levels, thereby limiting glucose transport.

### Milciclib limits glucose consumption by inhibiting CDK7

**Fig. 5:**
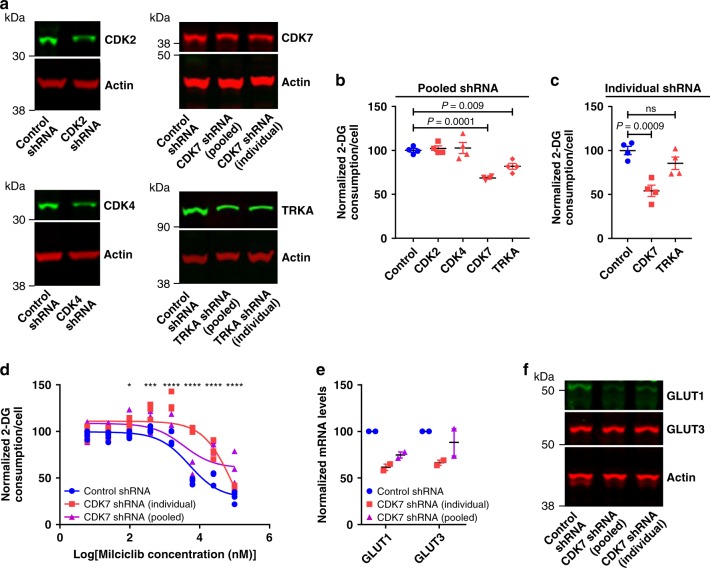
CDK7 promotes glucose consumption in H460 cells. **a** Immunoblots of lysate from H460 cells transfected with control shRNA or shRNA targeted against CDK2, CDK4, CDK7, and TRKA. *n* = 2. Glucose consumption in H460 cells transfected with control shRNA or **b** pooled or **c** individual shRNA separately targeted against CDK2, CDK4, CDK7, and TRKA. *n* = 4. *P* values determined by one-way ANOVA tests. **d** Glucose consumption dose response curves in H460 cells transfected with control shRNA or shRNA targeted against CDK7 and treated with Milciclib. *n* = 4. *P* values determined by a two-way ANOVA test. **e** mRNA levels from H460 cells transfected with control shRNA or pooled or individual shRNA targeted against CDK7. *n* = 2. **f** Immunoblots of lysate from H460 cells transfected with control shRNA or pooled or individual shRNA targeted against CDK7. *n* = 2. ns: not significant. *P < 0.05; ***P < 0.001; ****P < 0.0001. Data are plotted as mean ± SEM.

Known targets of Milciclib include CDK2, CDK4, CDK7, and TRKA^32,33^. We transfected H460 cells with a control shRNA or pooled shRNA targeting CDK2, CDK4, CDK7, or TRKA, which decreased the expression of their respective protein targets by 34.4 ± 2.7%, 44.8 ± 4.6%, 42.3 ± 0.9%, and 43.5 ± 2.8% compared to the control shRNA (Fig. 5a). Knockdown of CDK7 and TRKA significantly decreased glucose consumption to 68.5 ± 1.1% and 81.8 ± 3.3% of control values, respectively, while knockdown of CDK2 and CDK4 had no significant effect on glucose consumption (102.1 ± 3.0% and 102.7 ± 6.3% of control values, respectively; Fig. 5b). To further evaluate these results, H460 cells were transfected with a control shRNA or with individual shRNA targeting CDK7 or TRKA. Both constructs decreased the expression of their respective protein target (CDK7: 45.6 ± 1.3% decrease, TRKA: 42.1 ± 7.2% decrease; Fig. 5a), but only the shRNA targeting CDK7 significantly decreased cellular glucose consumption to 54.2 ± 6.3% of control values (Fig. 5c). CDK7 knockdown in H460 cells also decreased the ability of Milciclib to inhibit glucose consumption and cell growth (normalized cell numbers of H460 cells transfected with a control shRNA, pooled CDK7 shRNA, or individual CDK7 shRNA and treated with Milciclib: 63.5 ± 1.6, 71.8 ± 1.8, and 87.4 ± 3.9, respectively; Fig. 5d; Supplementary Fig. 7). CDK7 siRNA transfected into H460 cells decreased CDK7 protein levels by 52.8 ± 1.8% and yielded similar results (Supplementary Fig. 8). CDK7 knockdown in H460 cells significantly decreased GLUT1 but not GLUT3 mRNA and protein levels suggesting that CDK7 regulates GLUT1 mRNA and protein levels (Fig. 5e, f, Supplementary Fig. 8**;** GLUT1 mRNA levels: 31.9 ± 4.3% decrease, GLUT1 protein levels: 61.9 ± 5.8% decrease, averaged across both shRNA sequences). CDK2 and CDK4 knockdown did not significantly affect GLUT1 proteins levels but TRKA knockdown decreased GLUT1 protein levels by 13 ± 1.5%, averaged across both shRNA sequences (Supplementary Fig. 9). These data indicate that CDK7 knockdown and Milciclib treatment both inhibit glucose consumption and that CDK7 and Milciclib regulate glucose consumption through the same pathway. Combined with studies showing that Milciclib directly inhibits CDK7 (ref. ^32^), this data suggests that Milciclib blocks glucose consumption in H460 cells by inhibiting CDK7. Given the variable protein knockdown efficiencies and the potential that different proteins require different levels of depletion before a phenotypic effect can be observed, we cannot conclude from this data whether other known or unknown Milciclib targets also regulate glucose consumption.

Despite repeated attempts, the knockdown efficiency of CDK7 we were able to achieve in H460 cells was moderate, possibly due to the essential nature of this protein for cancer and other dividing cell populations^39,40^. The same studies suggest that CDK7 knockout cells would be unlikely to grow. To provide further evidence that CDK7 regulates glucose consumption in H460 cells, we tested two additional CDK7 inhibitors. THZ1 inhibits CDK7 with additional activity against CDK12 (ref. ^41^). LDC4297 inhibits CDK7 with additional activity against CDK2 and CDK5 (ref. ^42^). Notably, although Milciclib, THZ1, and LDC4297 all have additional targets beyond CDK7, none of these additional targets are shared between all three inhibitors. Both THZ1 and LDC4297 decreased glucose consumption in H460 but not A549 or HCC827 cells despite blocking cell growth in all three cell lines (Supplementary Fig. 10a). CDK7 knockdown in H460 cells reduced the ability of THZ1 and LDC4297 to block glucose consumption, suggesting that THZ1 and LDC4297 decrease glucose consumption by inhibiting CDK7 (Supplementary Fig. 10b, c). Consistent with a mechanism in which CDK7 inhibition blocks glucose transport, THZ1 significantly decreased GLUT1 mRNA and protein levels by 42.9 ± 4.4% and 40.7 ± 1.3%, respectively (Supplementary Fig. 11). LDC4297 significantly decreased GLUT1 mRNA and protein levels by 71.7 ± 8.3% and 39.2 ± 2.4%, respectively (Supplementary Fig. 11). Milciclib, THZ1, and LDC4297 did not decrease GLUT1 mRNA levels in HCC827 cells (Supplementary Fig. 12). Overall this data provides additional evidence that CDK7 regulates the expression of GLUT1 mRNA and protein levels.

CDK7 may promote HIF1α transcriptional activity in chronic lymphocytic leukemia cell lines, and the expression of GLUT1, GLUT3, Hexokinase 1, and Hexokinase 2 is regulated by HIF1α^43,44^. Our experiments were conducted at atmospheric oxygen concentrations, and HIF1α protein levels are undetectable in H460 cells and were not induced by Milciclib (Supplementary Fig. 13a). Additionally, Milciclib had no consistent effect on HIF1α-regulated gene products, repressing the expression of GLUT1, GLUT3, Hexokinase 1, Hexokinase 2, and Ceruloplasmin, while having no effect on the expression of SERPINE1 and inducing the expression of VEGFA (Fig. 4a and Supplementary Fig. 13b). These data provide evidence that Milciclib does not repress GLUT1, GLUT3, Hexokinase 1, and Hexokinase 2 mRNA levels through HIF1α regulation.

### PI3K signaling promotes glucose consumption through CDK7

CDK7 is a CDK-activating kinase (CAK), and as a subunit of the transcription factor TFIIH, it promotes various steps in transcription by phosphorylating Ser5 in the C-terminal domain (CTD) of the Rpb1 subunit of RNA polymerase II^45–47^. Since Milciclib, THZ1, and LDC4297 decrease GLUT1 mRNA levels (Fig. 4a**;** Supplementary Fig. 11), we focused on a potential mechanism through which CDK7 regulates GLUT1 transcription.

**Fig. 6:**
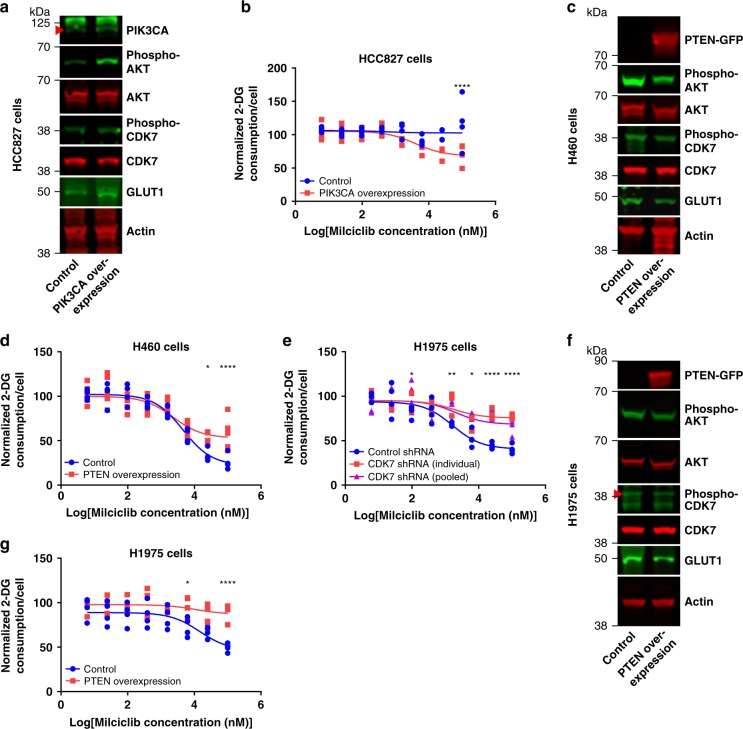
PIK3CA activates CDK7 to promote glucose consumption. **a** Immunoblots from HCC827 cells transfected with a control or PIK3CA overexpression plasmid. *n* = 2. **b** Glucose consumption dose response curves of HCC827 cells transfected with a control or PIK3CA overexpression plasmid and treated with Milciclib. *n* = 4. P values determined by a two-way ANOVA test. **c** Immunoblots from H460 cells transfected with a control or PTEN overexpression plasmid. *n* = 2. **d** Glucose consumption dose response curves of H460 cells transfected with a control or PTEN overexpression plasmid and treated with Milciclib. Control: *n* = 4, PTEN overexpression: *n* = 6. *P* values determined by a two-way ANOVA test. **e** Glucose consumption dose response curves in H1975 cells transfected with control shRNA or shRNA targeted against CDK7 and treated with Milciclib. *n* = 4 except *n* = 3 for CDK7 shRNA (individual). *P* values determined by a two-way ANOVA test. **f** Immunoblots of lysate from H1975 cells transfected with a control or PTEN overexpression plasmid. *n* = 2. **g** Glucose consumption dose response curves of H1975 cells transfected with a control or PTEN overexpression plasmid and treated with Milciclib. *n* = 4. *P* values determined by a two-way ANOVA test. *P < 0.05; **P < 0.01; ****P < 0.0001.

Studies suggest that phosphorylation of CDK7 at Thr170 can stabilize the TFIIH complex and selectively activate the CTD kinase activity of CDK7, and that the PI3K pathway may regulate phosphorylation at this site^47,48^. H460 cells have an activating E545K mutation in the catalytic PI3K subunit PIK3CA^30^. H460 cells are the only cell line among the three that we studied with a genetic alteration in the PI3K pathway^30^ and the only cell line in which Milciclib, THZ1, and LDC4297 block glucose consumption (Fig. 3c, Supplementary Fig. 10a), suggesting a potential role for the PI3K-signaling pathway in regulating glucose consumption through CDK7. Neither Milciclib, THZ1, nor LDC4297 inhibit glucose consumption in HCC827 cells (Fig. 3c, Supplementary Fig. 10a). However overexpression of wild-type PIK3CA in HCC827 cells increased phospho-AKT levels by 244 ± 30%, increased phospho-CDK7 levels by 48.3 ± 3.7%, increased GLUT1 levels by 85.0 ± 0.1%, and sensitized HCC827 cells to the inhibitory effect of Milciclib, THZ1, and LDC4297 on glucose consumption without significantly affecting how much Milciclib inhibits cell growth (normalized cell numbers of control and PIK3CA overexpressing HCC827 cells treated with Milciclib: 66.1 ± 1.6% and 72.1 ± 2.4%, respectively; Fig. 6a, b, Supplementary Fig. 14). The phosphatase and tensin homolog (PTEN) protein opposes the activity of PIK3CA^49^. Overexpression of PTEN in H460 cells decreased phospho-AKT levels by 55.5 ± 5.8%, decreased phospho-CDK7 levels by 38.7 ± 4.8%, decreased GLUT1 levels by 16.0 ± 1%, and blocked the inhibition of glucose consumption by Milciclib, THZ1, and LDC4297 without significantly affecting how much Milciclib inhibits cell growth (normalized cell numbers of control and PTEN-overexpressing H460 cells treated with Milciclib: 70.4 ± 4.1% and 69.9 ± 4.1%, respectively; Fig. 6c, d, Supplementary Fig. 15a). Glucose consumption in HCC827 cells is not sensitive to Milciclib (Fig. 3c) and overexpression of PTEN in HCC827 cells had no significant effect on the ability of Milciclib to inhibit glucose consumption despite decreasing phospho-AKT levels by 61.9 ± 5.9% (Supplementary Fig. 15b, c).

To further test our finding that aberrant PIK3CA activity promotes glucose consumption through CDK7, we evaluated H1975 cells, which have an activating G118D mutation in PIK3CA^30^. Similar to H460 cells (Fig. 3c, Supplementary Fig. 10a), Milciclib, THZ1, and LDC4297 reduced glucose consumption and cell growth in H1975 cells (Fig. 6e, Supplementary Fig. 16a). Individual or pooled shRNA constructs targeting CDK7 decreased CDK7 protein levels by 43.7 ± 0.2% and 41.1 ± 6.2%, GLUT1 mRNA levels by 43.1 ± 1.1% and 24.6 ± 0.9%, GLUT1 protein levels by 51.7 ± 2.7% and 46.0 ± 12.6%, and glucose consumption by 34.6 ± 4.1% and 35.3 ± 5.9%, respectively, and blocked Milciclib, THZ1, and LDC4297 from inhibiting glucose consumption in H1975 cells (Fig. 6e, Supplementary Fig. 16). Individual and pooled shRNA constructs targeting CDK7 also blocked Milciclib from inhibiting cell growth in H1975 cells (normalized cell numbers of H1975 cells transfected with a control shRNA, an individual CDK7 shRNA, or a pooled CDK7 shRNA and treated with Milciclib: 61.0 ± 5.0%, 96.3 ± 4.4%, and 86.1 ± 6.9%, respectively). CDK7 siRNA decreased CDK7 protein levels by 75 ± 8% in H1975 cells and yielded similar results (Supplementary Fig. 17). Milciclib, THZ1, and LDC4297 also significantly decreased GLUT1 mRNA and protein levels in H1975 cells (Supplementary Fig. 18). Consistent with a role for mutant PIK3CA in regulating glucose consumption through CDK7 in H1975 cells, overexpression of PTEN in these cells decreased phospho-AKT levels by 27.4 ± 1.6%, decreased phospho-CDK7 levels by 34.4 ± 1.5%, decreased GLUT1 levels by 46.5 ± 10.9%, and desensitized the cells to the inhibitory effects of Milciclib, THZ1, and LDC4297 on glucose consumption without significantly affecting how much Milciclib inhibits cell growth (normalized cell numbers of control or PTEN-overexpressing H1975 cells treated with Milciclib: 64.5 ± 4.5% and 66.3 ± 1.8%, respectively; Fig. 6f, g, Supplementary Fig. 19). These data fit a model in which mutant activated PIK3CA promotes enhanced glucose consumption in NSCLC cancer cells in part through the activation of CDK7. However, insofar as PI3K signaling regulates many different proteins and pathways^50^, we cannot rule out pleiotropic effects of PIK3CA or PTEN overexpression that may contribute to our results. For example, one protein known to regulate glucose consumption downstream of PI3K signaling is thioredoxin interacting protein (TXNIP)^51–54^. Although we do not identify significant changes in TXNIP protein levels in HCC827 cells that overexpress PIK3CA, in H460 cells that overexpress PTEN, and in H460 cells treated with Milciclib (Supplementary Fig. 20), we cannot rule out a role for TXNIP in our system.

### PKCι activates CDK7 to promote glucose consumption

**Fig. 7:**
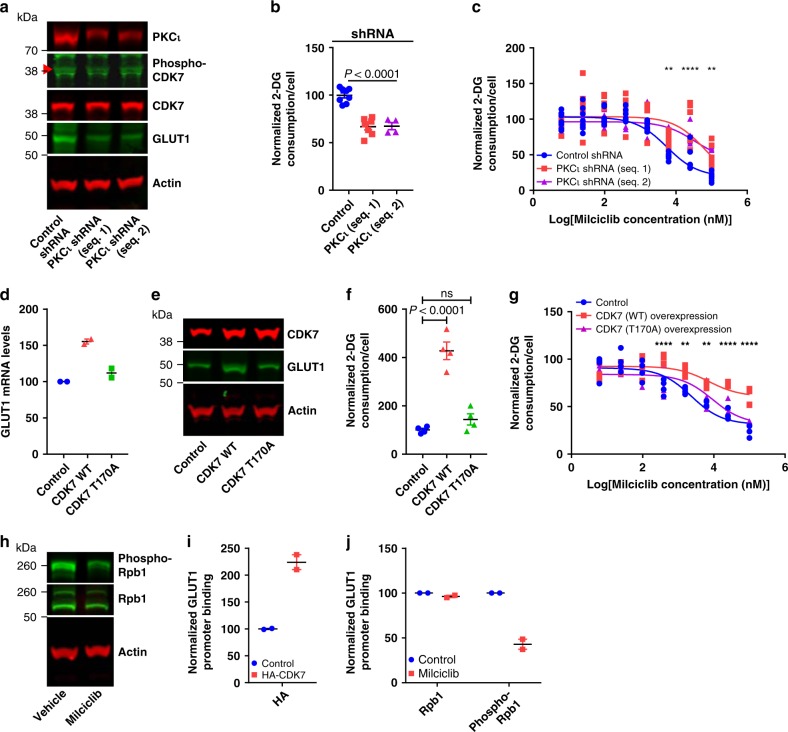
PKCι activates CDK7 to promote glucose consumption. **a** Immunoblots from H460 cells transfected with control shRNA or shRNA targeted against PKCι. Seq. 1 and 2 represent different shRNA sequences. *n* = 2. **b** Glucose consumption in H460 cells transfected with control shRNA or shRNA targeted against PKCι. Control: *n* = 8; Seq. 1: *n* = 7; Seq. 2: *n* = 4. *P* value determined by a one-way ANOVA test. **c** Glucose consumption dose response curves in H460 cells transfected with control shRNA or shRNA targeted against PKCι and treated with Milciclib. Control: *n* = 8; Seq. 1: *n* = 7; Seq. 2: *n* = 4. *P* values determined by a two-way ANOVA test. **d** GLUT1 mRNA levels from H460 cells transfected with a control, wild-type (WT) CDK7, or T170A mutant CDK7 overexpression plasmid. *n* = 2. **e** Immunoblots from H460 cells transfected with a control, WT CDK7, or T170A mutant CDK7 overexpression plasmid. *n* = 2. **f** Glucose consumption in H460 cells transfected with a control, WT CDK7, or T170A mutant CDK7 overexpression plasmid. *n* = 4. *P* values determined by a one-way ANOVA test. **g** Glucose consumption dose response curves in H460 cells transfected with a control, WT CDK7, or T170A mutant CDK7 overexpression plasmid and treated with Milciclib. *n* = 4. *P* values determined by a two-way ANOVA test. **h** Immunoblots from H460 cells treated with vehicle or Milciclib (10 μM). *n* = 2. **i** HA-CDK7 levels on the GLUT1 promoter in H460 cells transfected with a control or HA-CDK7 overexpression plasmid. *n* = 2. **j** Rpb1 and phospho-Rpb1 levels on the GLUT1 promoter in H460 cells treated with vehicle or Milciclib (10 μM). *n* = 2. ns: not significant. **P < 0.01; ****P < 0.0001. Data are plotted as mean ± SEM.

PKCι phosphorylates Thr170 on CDK7 in vitro, can associate with CDK7 in cells, and is activated downstream of PI3K signaling^48,55–58^. Transfection of H460 cells with two different shRNA targeted against PKCι decreased PKCι protein levels by 54.8 ± 3.4% and 52.2 ± 2.8%, phospho-CDK7 levels by 47.1 ± 8.8% and 42.4 ± 3.3%, GLUT1 protein levels by 38.6 ± 3.3% and 37.5 ± 3.0%, and glucose consumption by 33.6 ± 3.5% and 32.9 ± 3.8%, respectively, compared to cells transfected with a control shRNA (Fig. 7a, b). Transfection of H1975 cells with these same shRNA yielded better PKCι knockdown (by 76.8 ± 3.8 and 80.7 ± 5.4) but otherwise similar results (Supplementary Fig. 21a, b). Additionally, PKCι knockdown blocked the ability of Milciclib, THZ1, and LDC4297 to inhibit glucose consumption in both cell lines (Fig. 7c, Supplementary Fig. 21c, 22). PKCι knockdown had no significant effect on the ability of Milciclib to inhibit cell growth in H460 cells (normalized cell numbers of H460 cells transfected with control shRNA, expressing one PKCι shRNA sequence, and expressing a second PKCι shRNA sequence: 73.0 ± 2.2%, 67.5 ± 5.1%, and 76.4 ± 10.5%, respectively). Overexpression of wild-type CDK7 but not T170A phospho-mutant CDK7 in H460 cells induced GLUT1 mRNA levels by 55.4 ± 3.5%, GLUT1 protein levels by 50.0 ± 12%, and glucose consumption by 428 ± 37% (Fig. 7d–f). Overexpression of wild-type and T170A CDK7 in H1975 cells yielded similar results (Supplementary Fig. 23a–c). Overexpression of wild-type CDK7 also desensitized H460 and H1975 cells to the inhibitory effect of Milciclib, THZ1, and LDC4297 on glucose consumption but overexpression of a T170A phospho-site mutant did not (Fig. 7g, Supplementary Figs. 23 and 24). Overexpression of wild-type CDK7 in H460 cells also decreased the ability of Milciclib to block cell growth although this result did not reach statistical significance (normalized cell numbers of H460 cells transfected with control, wild-type CDK7, and T170A CDK7 and treated with Milciclib: 86.4 ± 9.0%, 101.1 ± 8.7%, and 81.5 ± 7.7%, respectively). These data provide evidence that PKCι regulates the phosphorylation of CDK7 at Thr170 to promote glucose consumption.

CDK7 phosphorylation at Thr170 promotes its ability to phosphorylate the CTD of the Rpb1 subunit of RNA Polymerase II and regulate multiple transcriptional steps^45–47^. Milciclib, THZ1, and LDC4297 all decreased phospho-Ser5 Rpb1 levels in H460 and H1975 cells (Fig. 7h, Supplementary Figs. 25a and 26). Milciclib had no effect on phospho-Ser5 Rpb1 levels in A549 and HCC827 cells (Supplementary Fig. 25b). CDK7 binds to the GLUT1 promoter in H460 cells (Fig. 7i). Milciclib, THZ1, and LDC4297 also decreased phospho-Ser5 Rpb1 levels on the GLUT1 promoter in H460 and H1975 cells (Fig. 7j, Supplementary Figs. 27 and 28). This suggests that inhibition of CDK7-dependent Rpb1 phosphorylation may explain the decrease in GLUT1 mRNA levels.

**Fig. 8:**
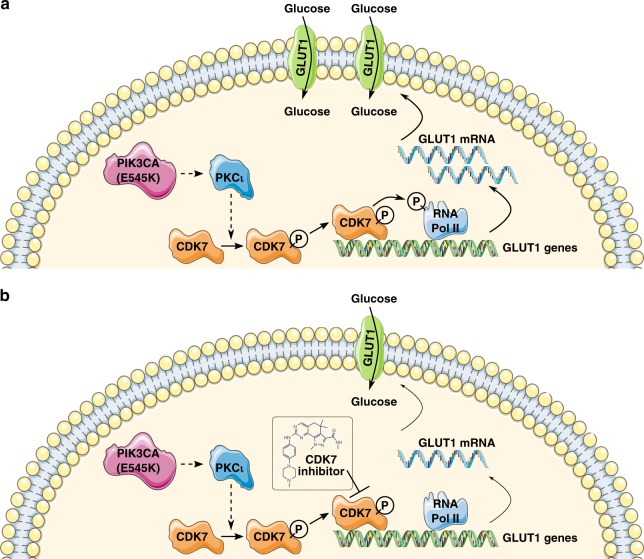
CDK7 activates glucose transport in certain NSCLC. **a** Activated PIK3CA signals through PKCι to induce CDK7 phosphorylation. Phosphorylation of CDK7 activates it to phosphorylate RNA Polymerase II (RNA Pol II). This leads to transcription of GLUT1 mRNA, expression of the GLUT1 transporter, and transport of glucose across the cell membrane. **b** CDK7 inhibitor treatments block CDK7 activity, decreasing the phosphorylation of RNA Pol II. This leads to a decrease in GLUT1 mRNA and protein levels, and less glucose transport. Dotted lines are meant to represent the regulation of one protein by another but not necessarily a direct, linear interaction between the proteins.

Collectively, our data support the following model (Fig. 8): Oncogenic mutant PIK3CA activates the PI3K pathway, leading to elevated PKCι activity, and increased CDK7 phospho-Thr170 levels. Phospho-CDK7 is activated to phosphorylate Ser5 in the CTD of the Rpb1 subunit of RNA Polymerase II on the promoter of various genes, including the GLUT1 gene, leading to increased GLUT1 mRNA and protein levels, and increased glucose transport. The interactions between PIK3CA, PKCι, and CDK7 may be direct or indirect and not necessarily linear. Small molecule inhibitors such as Milciclib, THZ1, and LDC4297 can interrupt this signaling cascade by inhibiting CDK7, thereby decreasing Ser5 CTD phosphorylation at the GLUT1 promoter, leading to decreased GLUT1 mRNA and protein levels, and decreased glucose transport. Although literature suggests that phosphoinositide-dependent kinase 1 (PDPK1)^48,59,60^, a protein downstream of PIK3CA signaling, phosphorylates PKC isozymes, it remains possible that PKCι regulates CDK7 independent of PIK3CA. Additional studies will be required to further evaluate and develop this model.

## Discussion

Despite the clear importance of glucose consumption in health and disease, identifying new regulators of glucose consumption remains a slow process. Although some have been identified through unbiased analyses, most such regulators are discovered through targeted studies^54,61^, and currently none of these approaches are high-throughput. Here, we describe a high-throughput assay for measuring cellular glucose consumption. We anticipate that this assay, when coupled to available chemical or genetic libraries, will enable the unbiased identification of pathways and proteins that regulate glucose consumption.

We identify that CDK7 inhibition decreases GLUT1 mRNA levels, GLUT1 protein levels, and glucose transport. Glucose transport across the cell membrane alone or in combination with glucose phosphorylation is a major rate-limiting step for glycolysis across multiple systems^62^. Consistent with this, cancer cells commonly regulate glucose metabolism by controlling glucose transport^4–7^. Our results add additional data to support the important role for glucose transporters in regulating cancer cell glucose consumption.

Previous studies suggest various mechanisms through which enhanced PI3K signaling drives glucose consumption^9,63^. Here, we present data to support an alternative mechanism: Mutant PIK3CA, possibly through the activation of PKCι, activates CDK7, leading to increased mRNA expression of GLUT1; increased protein levels of GLUT1; and increased glucose transport. This model suggests various proteins that could potentially be targeted to limit glucose consumption in cancer cells with increased PI3K activity. Moreover, the regulation of glucose consumption by CDK7 provides an additional connection between cell cycle regulation and cellular metabolism, building on recent work demonstrating roles for CDK6 and CDK8 in regulating glucose consumption and glycolysis^61,64^. These studies combined with ours suggest that cyclin-dependent kinases may represent key and potentially targetable cellular nodes that co-regulate metabolism and cell cycle progression, possibly functioning to ensure that cycling cells have adequate nutrients to complete cell division.

We screened 3555 drugs against three NSCLC lung cancer cell lines with different genetic drivers and failed to identify any drug that decreases glucose consumption in all three cell lines by > 50%, our threshold for classifying that a small molecule inhibits glucose consumption. Although this could be due to how we set this threshold, the data may also suggest that different genetic drivers use different mechanisms to promote glucose consumption. Further experiments across additional cell lines with similar or different engineered or endogenous driver mutations would be required to make this conclusion. However, if this conclusion were proven true, it would suggest that the underlying driver mutations of a patient’s tumor would have to be known before that patient could be treated with a small molecule to inhibit glucose consumption in that tumor.

Efforts to target cancer cell metabolism have focused primarily on identifying direct inhibitors of metabolic enzymes whose functions are critical for cancer cell growth and survival^65,66^. However, except in rare cases, these same enzymes are present in healthy tissue throughout the body and most clinical metabolic inhibitors target only one pathway— nucleotide biosynthesis—suggesting a potentially unique therapeutic window for directly inhibiting enzymes in this pathway in cancer^65,66^. Targeting glucose consumption with small molecule inhibitors of enzymes in this pathway has proven challenging^17^, likely due to a requirement for glucose in the function of many organ systems. Here, we show that CDK7 inhibitors block glucose consumption downstream of an activating PIK3CA mutation and that in mice, Milciclib does not affect glucose consumption in healthy tissue. Thus Milciclib may be a more selective inhibitor of glucose consumption in cancer than other available small molecules. Because we identify distinct inhibitors of glucose consumption in genetically different cancer cell lines, we anticipate that the efficacy of many of these inhibitors will depend on cancer-specific genetic alterations and will also be selective for cancer over healthy cells.

We show that CDK7 overexpression and paradoxically CDK7 knockdown both decrease the sensitivity of H460 and H1975 cells to CDK7 inhibitors. Haploinsufficiency screens in yeast and mammalian cells to identify targets of cytotoxic drugs suggest that knockdown of CDK7 should increase the sensitivity of H460 and H1975 cells to the CDK7 inhibitors^67^. We cannot explain this paradox. One potential explanation is that another protein compensates for CDK7 in its absence. Various other proteins have been identified that can phosphorylate Rpb1 Ser5, including other cyclin-dependent kinases^46,68^. However, CDK7 has been suggested to contribute to multiple steps of transcription^46,69^. Which of these steps are necessary for GLUT1 transcription and which can or cannot be compensated for has yet to be established. An alternative potential explanation is that CDK7 only regulates glucose consumption during a certain stage of the cell cycle which CDK7 knockdown decreases. During CDK7 knockdown, the number of cells in the specific stage of the cell cycle during which CDK7 regulates glucose consumption would be decreased and therefore the ability of CDK7 inhibitors to limit glucose consumption would be blocked. Additional work will be required to determine which if any of these explanations holds merit.

We also show that overexpression of the T170A phospho-mutant CDK7 does not decrease the sensitivity of H460 and H1975 cells to CDK7 inhibitors. Phosphorylation of CDK7 at Thr170 stabilizes the TFIIH complex, which phosphorylates RNA Polymerase II to promote proper transcription of genes^47,70^. In our experiments, the TFIIH complexes formed from the CDK7 T170A phospho-mutant are likely less stable than the TFIIH complexes formed from wild-type CDK7, which may explain why the CDK7 T170A phospho-mutant has no effect on the ability of CDK7 inhibitors to alter GLUT1 mRNA levels or glucose consumption. Insofar as CDK7 binds to multiple other proteins to form the TFIIH complex^71^, this explanation requires that these other proteins are expressed at higher levels than CDK7 in the cells or that CDK7 overexpression induces the expression of these proteins.

In our high-throughput screen, we treated cells with small molecules for 24 h before removing the media and measuring glucose consumption in PBS without drug, so we are unlikely to have identified small molecules that directly inhibit the proteins that transport or phosphorylate glucose. Instead the compounds we identified likely modulate signaling pathways and cellular processes that either directly or indirectly regulate glucose consumption. However, insofar as our high-throughput assay specifically measures the combined activities of the first two steps in glucose consumption—glucose transport and phosphorylation—we know that every drug we identify decreases the activity of one or both of these steps.

We show that Milciclib decreases H460 cell xenograft ^18^F-FDG consumption, possibly suggesting that decreased tumor ^18^F-FDG consumption measured by PET in patients could be used as a pharmacodynamic biomarker of CDK7 inhibitors. However, we also show that Milciclib, THZ1, and LDC4297 decrease growth in HCC827 and A549 cells without inhibiting glucose consumption, suggesting that in certain genetic backgrounds, CDK7 inhibition can block cell growth without altering glucose consumption. If this is the case, a positive change in ^18^F-FDG consumption following treatment with a CDK7 inhibitor may suggest effective targeting, but no change in ^18^F-FDG consumption would have little predictive value.

Pathological glucose consumption is an important phenotype in cancer, but there are many diseases in which glucose consumption is affected and in which identifying modulators of glucose consumption could have value. Thus, we anticipate that our high-throughput screening assay will have utility across a variety of cell sources and systems.

## Methods

### Cell lines

A549 (CCL-185), NCI-H460 (HTB-177), NCI-H1975 (CRL-5908), and HCC827 (CRL-2868) cells were purchased from ATCC and cultured in RPMI supplemented with 10% (v/v) FBS, glutamine (4 mM), and penicillin–streptomycin (100 U mL^−1^). The cell lines were tested for mycoplasma contamination but were not authenticated. All cell lines were transduced with a plasmid that expresses nuclear-localized blue-fluorescent protein (FU-EBFP2-H2B-W; obtained from the UCLA Molecular Screening Shared Resource (MSSR)).

### Drug treatments

Individual drugs were purchased from Sigma-Aldrich, Selleck Chem, and Cayman Chemical and dissolved in DMSO. Cells were treated with drugs for 24 h unless otherwise noted. For those experiments that yielded dose response curves, cells were treated with drugs over the range of concentrations described in the figures. For those experiments in which only one drug concentration was tested, the following drug concentrations were used: Milciclib (10 µM), THZ1 (10 µM), LDC4297 (10 µM), Buparlisib (1 µM), Erlotinib (200 nM), and Cytochalasin B (10 µM; added to cells immediately prior to the addition of 2-DG or ^3^H-2-DG).

### 2-DG luminescence assay

Following vehicle or drug treatments, 2-DG consumption was measured using the Glucose Uptake-Glo kit (Promega Corporation) per the manufacturer’s protocol.

### ^3^H-2-DG consumption

Following vehicle or drug treatments, media was removed from cells, and the cells were washed (3×; 1× PBS). ^3^H-2-DG (1.25 mM, 5 µCi in 1x PBS) was added to the cells and the cells were incubated (30 min; 37 °C). The cells were washed (3×; cold 1× PBS), lysed in RIPA buffer, the lysate was added to scintillation fluid, and the radioactivity was measured on a scintillation counter. 2-DG accumulation was normalized to cell numbers.

### Compound libraries

The Selleck Chemicals kinase inhibitor library, the Prestwick FDA-approved drug library, the LOPAC collection, and the NIH clinical collection small molecule library were prepared and maintained by the UCLA MSSR.

### High-throughput 2-DG luminescence assay

A small molecule compound (1 mM in DMSO; 0.5 µL) or DMSO (0.5 µL) was pinned into 25 µL of growth media in wells of a 384-well plate and cells (0.26 × 10^6^ cells mL^−1^ in 25 µL of growth media) were dispensed into these wells using a Multidrop dispenser. The plates were incubated for 24 h at 37 °C, 5% CO_2_. After the 24 h incubation, the plates were washed (1x PBS containing 0.25% (w/v) BSA; 3×; 80 µL) and 2-DG (1.25 mM in 1× PBS containing 0.25% (w/v) BSA; 20 µL) was added using a BioTek EL406 Microplate washer/dispenser. The fluorescence in each well of the 384-well plate was imaged using an Image Express XL (10 min; 37 °C) and the plates were further incubated (10 min; 37 °C). Stop buffer (0.4 N HCl containing 2% (w/v) dodecyltrimethylammonium bromide; 6 µL) was added using the BioTek EL406 Microplate washer/dispenser, the plates were spun (2200 rpm; 1 min), shaken (900 rpm, 1 min), and spun again (2200 rpm, 1 min), and detection reagent (Glucose Uptake-Glo kit detection reagent diluted 1:1 in 1 M tris base; 40 µL) was added using a Multidrop dispenser. The plates were incubated at room temperature for 1 h, after which luminescence per well was measured on a PerkinElmer EnVision plate reader. For each well, luminescence was normalized to total cell numbers (determined from the fluorescent microscope image), and values for individual wells were normalized to the DMSO control for that row. Cytochalasin B (10 µM) was added to designated wells immediately prior to the addition of 2-DG. Control experiments in which 2-DG was not added were performed exactly the same except that 1× PBS without 2-DG was used in place of the 1X PBS with 2-DG (please see also Supplementary Table 2).

Those small molecules that inhibited glucose consumption by > 50% were rescreened in duplicate, and any small molecule that inhibited glucose consumption by > 3 standard deviations from the mean in this experiment was considered a validated inhibitor of glucose consumption.

### Cell growth

BFP+ cells were counted during the 2-DG luminescence assay using an Image Express XL. Normalized cell numbers were calculated as the ratio between the number of cells in wells treated with 10 μM Milciclib and the number of cells in wells treated with DMSO and reported as a percentage.

### Measurements of glucose and lactate media concentrations

Cells were incubated in media containing DMSO or Milciclib (10 μM) for 20 h, after which the media was changed into freshly prepared RPMI containing DMSO or Milciclib (10 μM). Four hours later, media (1 mL) was collected and analyzed, thus allowing us to measure media glucose consumption from 20 to 24 h post-Milciclib treatment. Glucose and lactate concentrations in the media were measured using a Bioprofile Basic Analyzer per the manufacturer’s protocol.

### ^18^F-FDG PET

H460 xenografts were implanted into the subcutaneous space of NOD *scid* gamma mice. When the tumors had reached ~0.05 cm^3^, mice were fasted overnight, anesthetized, ^18^F-FDG (~3 MBq) was injected through the tail vein, and one hour later the mice were imaged on a G8 PET/CT. Mice were treated with Milciclib (30 mg kg^−1^ in 0.5% carboxymethylcellulose; PO; BID) or vehicle (0.5% carboxymethylcellulose; PO; BID), and 24 h after the first treatment, imaged again with ^18^F-FDG PET. Analyses were conducted in the AMIDE software. Three-dimensional regions of interest (ROI) were drawn around the tumor and the mouse to measure total tumor activity and total injected dose, respectively, and these values were used to calculate the percent injected dose per cubic centimeter (%ID/cc) in the tumor. All mouse experiments complied with relevant ethical guidelines and were approved by the UCLA Animal Research Committee.

### qRT-PCR

RNA was isolated from H460 cells, 20 and 24 h after treatment with DMSO or Milciclib (10 μM) or 16 h after treatment with Deferoxamine (100 μM) using the GeneJET RNA purification kit (Thermo Fisher) per the manufacturer’s protocol. Reverse transcription and quantitative real-time PCR was conducted using ProtoScript II First Strand cDNA Synthesis Kit (New England BioLabs) and PowerUp SYBR Green Master Mix (Thermo Fisher), respectively, following the manufacturer’s protocol and using the following primers:

GLUT1 (SLC2A1) forward primer: GATTGGCTCCTTCTCTGTGG

GLUT1 (SLC2A1) reverse primer: TCAAAGGACTTGCCCAGTTT

GLUT3 (SLC2A3) forward primer: GTCTGAAGAGCTATGGCCGC

GLUT3 (SLC2A3) reverse primer: AACCGCTGGAGGATCTGCTT

Hexokinase 1 forward primer: GGACTGGACCGTCTGAATGT

Hexokinase 1 reverse primer: ACAGTTCCTTCACCGTCTGG

Hexokinase 2 forward primer: CAAAGTGACAGTGGGTGTGG

Hexokinase 2 reverse primer: GCCAGGTCCTTCACTGTCTC

Ceruloplasmin forward primer: CCCTGGAGAATGGATGCTCA

Ceruloplasmin reverse primer:CTAACATGCTTCCCACGGATATT

SERPINE1 forward primer: CACAAATCAGACGGCAGCACT

SERPINE1 reverse primer: CATCGGGCGTGGTGAACTC

VEGFA forward primer: AGGAGGAGGGCAGAATCATCA

VEGFA reverse primer: CTCGATTGGATGGCAGTAGCT

MAPK forward primer: CCTCAAGCCTTCCAACCTG

MAPK reverse primer: ATAATTTCTGGAGCCCTGTACC

All mRNA levels were normalized to MAPK mRNA levels.

### Immunoblots

Cell lysates from H460, A549, H1975, and HCC827 cells were prepared 24 h after treatment with DMSO, Milciclib (1 or 10 μM), THZ1 (10 μM), or LDC4297 (10 μM); 16 h after treatment with 1× PBS or Deferoxamine (100 μM); or 72 h after transfection with shRNA or an overexpression plasmid. Uncropped immunoblots are presented in Supplementary Fig. 29.

The following antibodies were used: HIF-1α (Novus Biologicals; NB100-105; 1:1000 dilution), GLUT1 (Millipore Sigma; 07-1401; 1:500 dilution), GLUT3 (Abcam; ab15311; 1:1000 dilution), Hexokinase 1 (Cell Signaling; C35C4; 1:1000 dilution), Hexokinase 2 (Cell Signaling; C64G5; 1:1000 dilution), CDK2 (Cell Signaling; 78B2; 1:1000 dilution), CDK4 (Cell Signaling; D9G3E; 1:1000 dilution), CDK7 (Cell Signaling; MO1; 1:1000 dilution), TRKA (Cell Signaling; 12G8; 1:1000 dilution), PIK3CA (Cell Signaling; C73F8; 1:1000 dilution), PTEN (Cell Signaling; 9559; 1:1000 dilution), Phospho-T170 CDK7 (Abcam; ab155976; 1:1000 dilution), Rpb1 NTD (Cell Signaling; D8L4Y; 1:1000 dilution), Phospho-S5 Rpb1 (Cell Signaling; D9N5I; 1:1000 dilution), PKCι (BD Biosciences; Clone 23; 1:1000 dilution), TXNIP (Cell Signaling; D5F3E; 1:1000 dilution), and Beta Actin (Imgenex; IMG-5142; 1:1000 dilution). Immunoblots were analyzed using an Odyssey Imaging System (LI-COR).

### FRET

H460 cells were transfected with the FLIPglu-700 FRET construct^36–38^ using Fugene HD per the manufacturer’s protocol. Two days post-transfection, FRET studies were performed 24 h after treatment with DMSO or Milciclib (10 μM) by monitoring emissions from the yellow fluorescent protein (YFP) and the cyan fluorescent protein (CFP) (535/40 and 480/30 nm wavelengths, respectively; signal separated by a 505 nm long-pass dichroic filter) in cells exposed to light at a wavelength that excites CFP (436/20b nm wavelength). Cells in 1X PBS were treated with glucose (10 mM) with or without Cytochalasin B (10 µM). Results were analyzed by plotting the ratio of the YFP and CFP signal and fitting this to an exponential decay curve.

### Plasmid overexpression

H460, HCC827, and H1975 cells were plated into wells of a six-well plate and transfected with control YFP, GLUT1, GLUT3, PTEN-GFP, PIK3CA, or HA-CDK7 plasmids using Fugene HD per the manufacturer’s protocol. Two days later, the cells were transferred to wells of a 384-well plate containing defined concentrations of the different inhibitors. Glucose consumption was measured 24 h later per the high-throughput method described above. The PIK3CA overexpression plasmid was a gift from Jean Zhao (Addgene plasmid #12522). The PTEN-GFP plasmid was a gift from Alonzo Ross (Addgene plasmid #13039). The HA-CDK7 plasmid was a gift from Matija Peterlin (Addgene plasmid # 14647). The GLUT and YFP plasmids were a gift from the Nathanson Lab (UCLA).

### Mutagenesis

The CDK7 T170A mutant was prepared using a Q5 Site-Directed Mutagenesis kit (New England BioLabs) according to the manufacturer’s protocol and using the following primers:

CDK7 T170A forward primer: TAGAGCTTATgcaCATCAGGTTG

CDK7 T170A reverse primer: TTGGGGCTCCCAAAAGAT

### shRNA and siRNA

Control shRNA, pooled shRNA, and individual shRNA constructs were obtained from the MSSR or Sigma Aldrich. Control siRNA and CDK7 siRNA were obtained from Dharmacon. H460 and H1975 cells were transfected with shRNA constructs and siRNA sequences using Fugene HD per the manufacturer’s protocol. Two days later, the cells were plated into wells of a 384-well plate containing various concentrations on each different inhibitor and 24 h later, glucose consumption was measured in high-throughput as described above.

### Chromatin immunoprecipitation (ChIP)

ChIP was performed on H460 cells transfected with a control YFP or CDK7 HA construct, or on H460 and H1975 cells 6 h after treatment with DMSO, Milciclib, THZ1, or LDC4297 (10 μM) using the SimpleChIP Plus ChIP kit (Cell Signaling) following the manufacturer’s protocol. The following antibodies were used: HA (Abcam; ab9110; 1:714 dilution), Rpb1 NTD (Cell Signaling; D8L4Y; 1:147 dilution), and phospho-Ser5 Rpb1 CTD (Cell Signaling; D9N5I; 1:50 dilution). qPCR was performed as previously described using the following primers:

GLUT1 (SLC2A1) forward primer: CCCTAGTGCACCGAAGTCAC

GLUT1 (SLC2A1) reverse primer: GTACCCGGCTGTAAGGCAAG

### Data analysis

All data are normalized to control knockdown, overexpression, or vehicle-treated cells except for the dose response curves. For the dose response curves, data is normalized to vehicle-treated cells for each genotype. Data were plotted, IC_50_ values were calculated, and statistics were performed using Graphpad Prism 7. All data are plotted as mean ± SEM.

### Statistical analyses

Pairwise two-sided *t* tests, and one-way and two-way ANOVA analyses with multiple comparison tests were performed. In all experiments except for the ^18^F-FDG PET experiment, measurements were made from distinct samples. In the ^18^F-FDG PET experiment, measurements were made on the same xenografts, before and after treating the mice with vehicle or Milciclib.

### Reporting summary

Further information on research design is available in the Nature Research Reporting Summary linked to this article.

## Supplementary information

Supplementary Information

Reporting Summary

## Data Availability

The data is available in the Article, Supplementary Information, or from the authors upon reasonable request.
