# Supplementary material for: A high-throughput screen identifies that CDK7 activates glucose consumption in lung cancer cells
Source: Nat Commun. 2019 Nov 29;10:5444. doi: 10.1038/s41467-019-13334-8 (PMC6884612; doi:10.1038/s41467-019-13334-8)
Supplement: Supplementary file 1 — Supplementary Information [file 41467_2019_13334_MOESM1_ESM.pdf]

Supplementary information

A high-throughput screen identifies that CDK7 activates glucose consumption in lung cancer cells

Chiara Ghezzi, Alicia Wong, Bao Ying Chen, Bernard Ribalet,

Robert Damoiseaux, Peter M. Clark

Supplementary Figures 1 – 29

Supplementary Tables 1 and 2

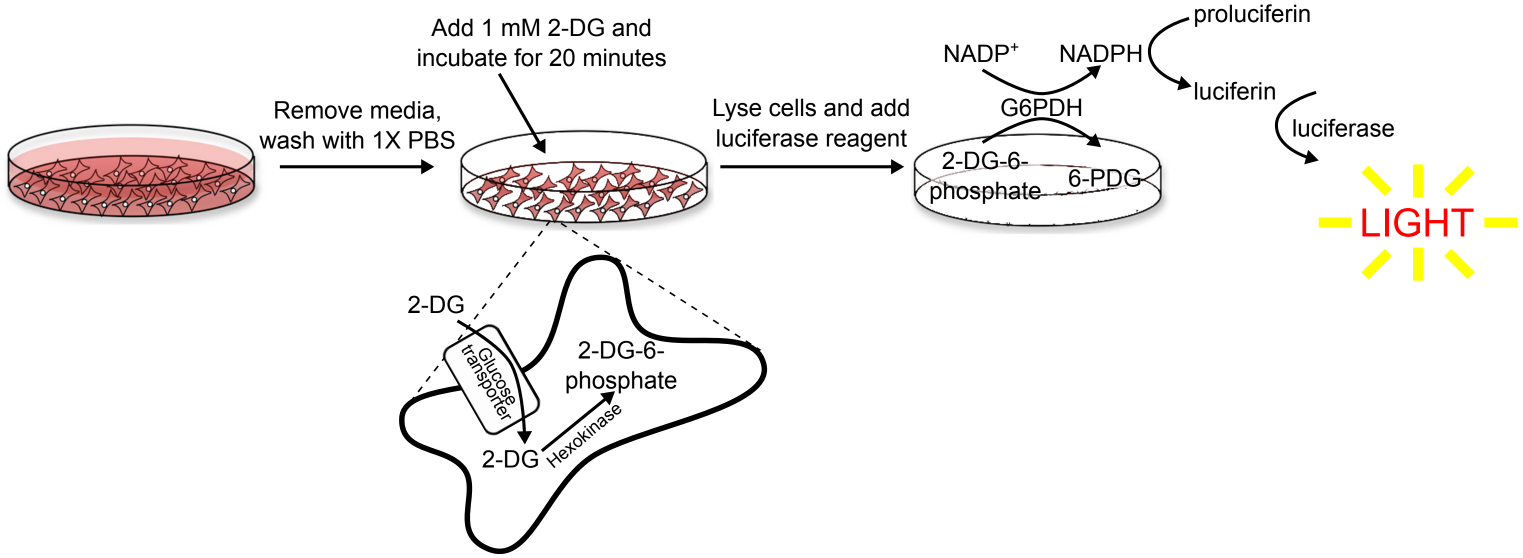

Supplementary Figure 1. Schematic of a luminescence-based glucose consumption assay. In adapting this general strategy into a high-throughput assay, we included the use of fluorescently labelled cells to enable automated cell counting by a fluorescent microscope.

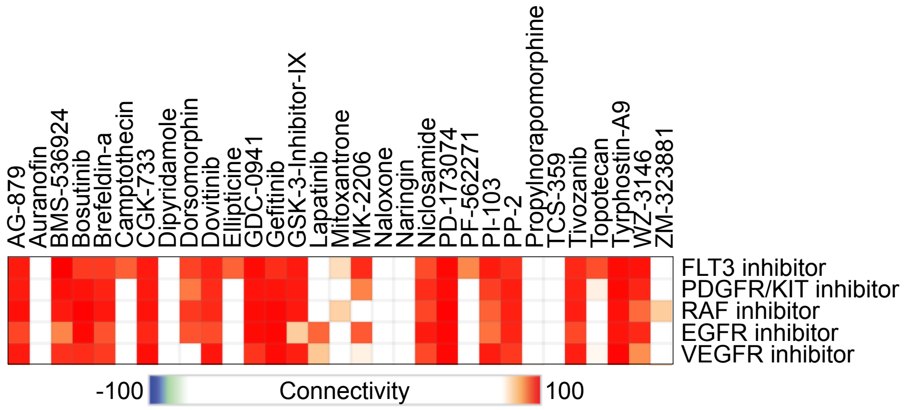

Supplementary Figure 2. Perturbagen classes connected to the small molecules identified as inhibitors of glucose consumption. Analysis performed using the Connectivity Map.

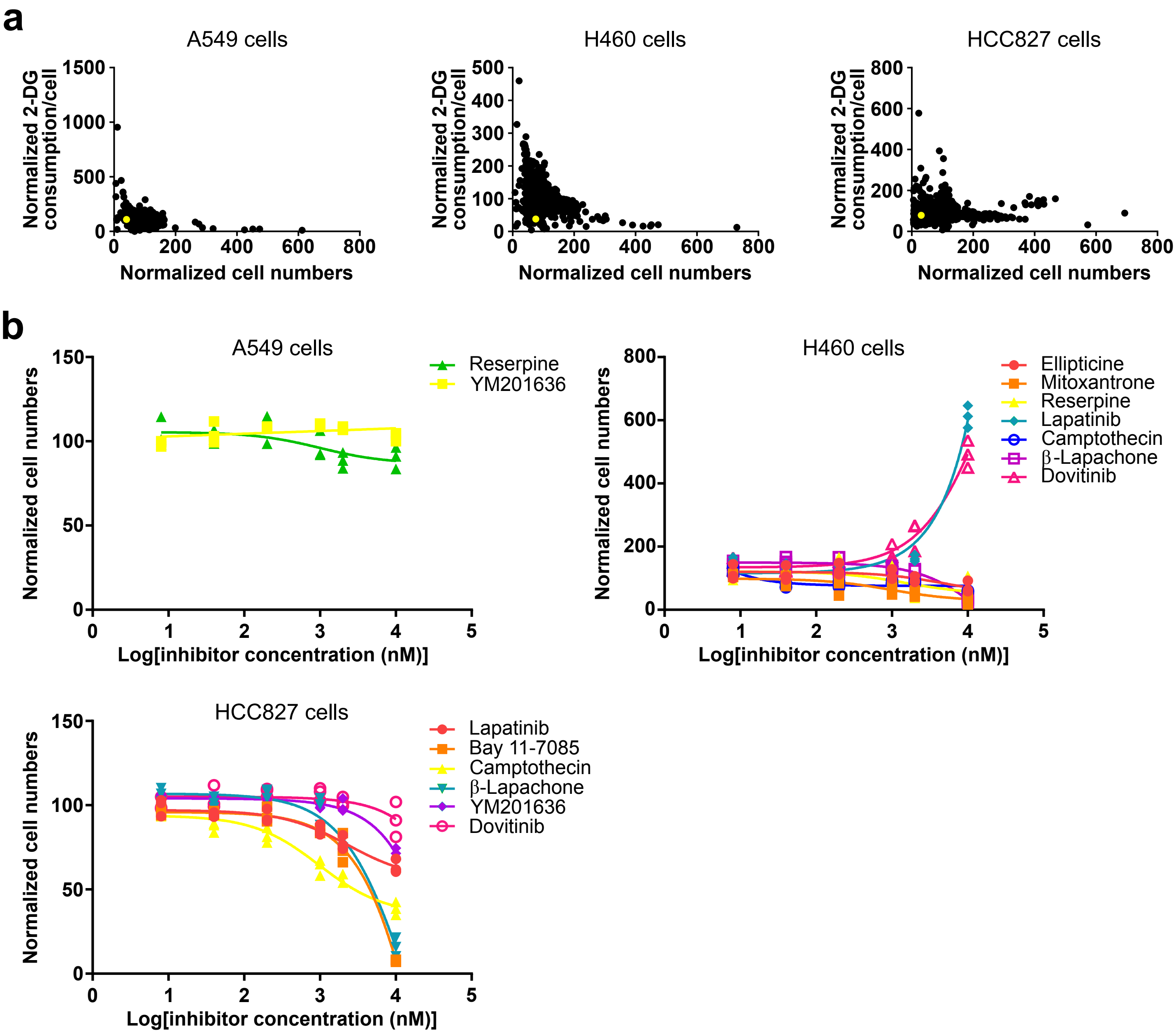

Supplementary Figure 3. Changes in cell growth and glucose consumption, 24 hours post-inhibitor treatment, are poorly correlated. a, Scatter plots of normalized 2-DG consumption/cell and normalized cell numbers in A549, H460, and HCC827 cells 24 hours after treatment with one of 3555 small molecule inhibitors. *n*=1. Milciclib is denoted as a yellow dot. **b,** Cell growth dose response curves for a subset of the compounds identified in the high-throughput screen (glucose consumption dose response curves for these compounds are displayed in **Figure 2d**). *n*=3 per concentration.

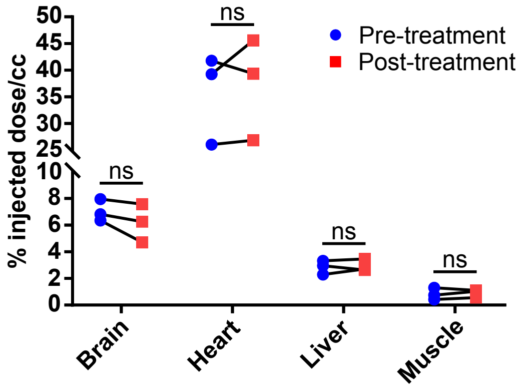

Supplementary Figure 4. Milciclib does not affect ^{18}F-FDG accumulation in the mouse brain, heart, liver, or muscle. ns: not significant. P values determined by paired t tests. *n*=3.

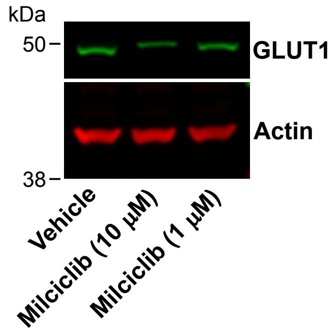

Supplementary Figure 5. Milciclib decreases GLUT1 protein levels. Immunoblots of lysate from H460 cells treated with vehicle or Milciclib. *n*=2.

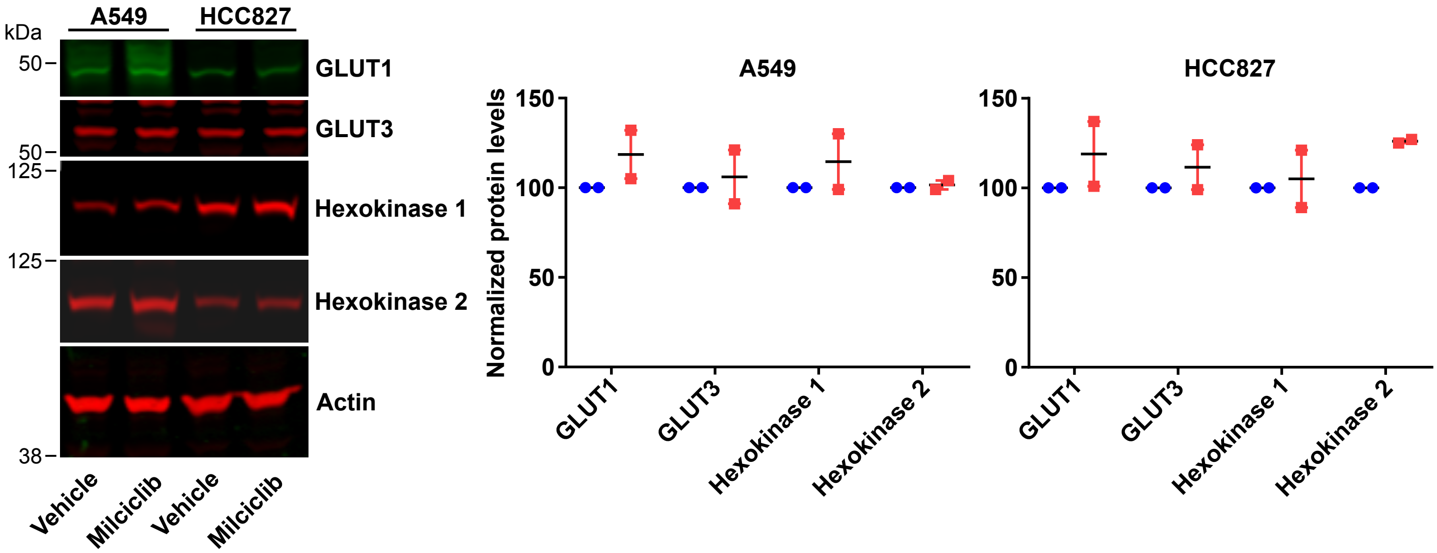

Supplementary Figure 6. Milciclib has no significant effect on or slightly increases GLUT1, GLUT3, Hexokinase 1, and Hexokinase 2 protein levels in A549 or HCC827 cells. Immunoblots (*left*) and quantification (*right*) of lysate from A549 and HCC827 cells treated with vehicle or Milciclib (10 μM). *n*=2. Data are plotted as mean ± SEM.

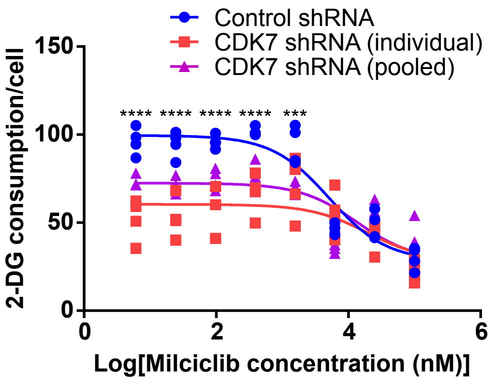

Supplementary Figure 7. CDK7 levels alter the ability of Milciclib to inhibit glucose consumption in H460 cells. Glucose consumption dose response curves in H460 cells transfected with control shRNA or shRNA targeted against CDK7 and treated with Milciclib. *n*=4. The data presented here is the same as in **Figure 5d** except that 2-DG consumption/cell has not been normalized to DMSO controls for each specific genetic perturbation. IC_{50} values for control shRNA, CDK7 shRNA (individual), and CDK7 shRNA (pooled) are 5 μM [95% confidence interval: 3 to 11 μM], 19 μM [95% confidence interval: 6 to 55 μM], and 14 μM [95% confidence interval: 3 to 78 μM], respectively. P values determined by a two-way ANOVA test. ***: P<0.001; ****: P<0.0001.

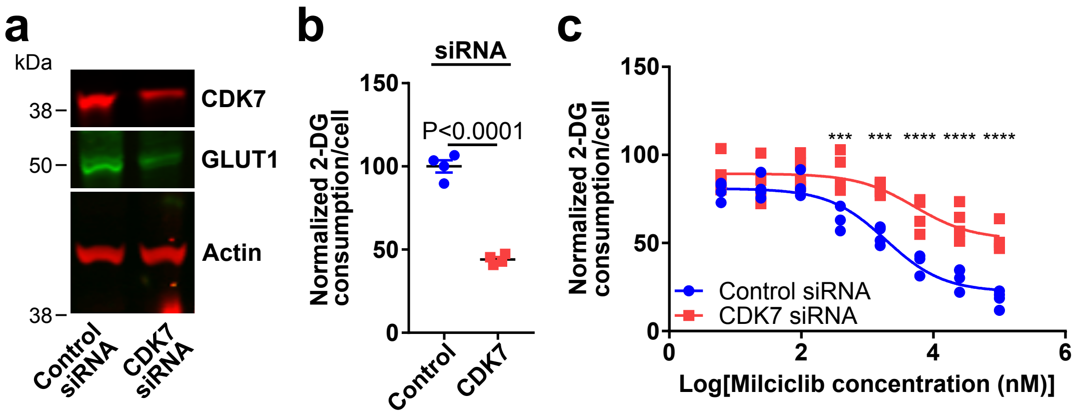

Supplementary Figure 8. CDK7 promotes glucose consumption in H460 cells. a, Immunoblots of lysate from H460 cells transfected with control siRNA or siRNA targeted against CDK7. *n*=2. **b,** Glucose consumption in H460 cells transfected with control siRNA or siRNA targeted against CDK7. *n*=4. P value determined by an unpaired t test. **c,** Glucose consumption dose response curves in H460 cells transfected with control siRNA or siRNA targeted against CDK7 and treated with Milciclib. *n* = 4. P values determined by a two-way ANOVA test. ***: P<0.001; ****: P<0.0001. Data are plotted as mean ± SEM.

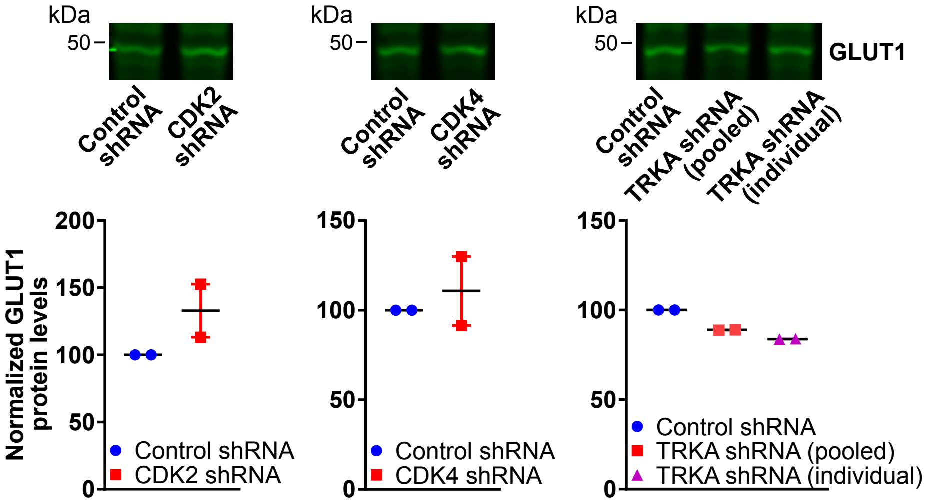

Supplementary Figure 9. CDK2 and CDK4 knockdown did not affect GLUT1 protein levels but TRKA knockdown decreased GLUT1 protein levels. Immunoblots of lysate from H460 cells transfected with control shRNA or shRNA targeted against CDK2, CDK4, and TRKA. Corresponding actin and CDK2, CDK4, and TRKA immunoblots for these samples are displayed in **Figure 5a**. *n*=2. Data are plotted as mean ± SEM.

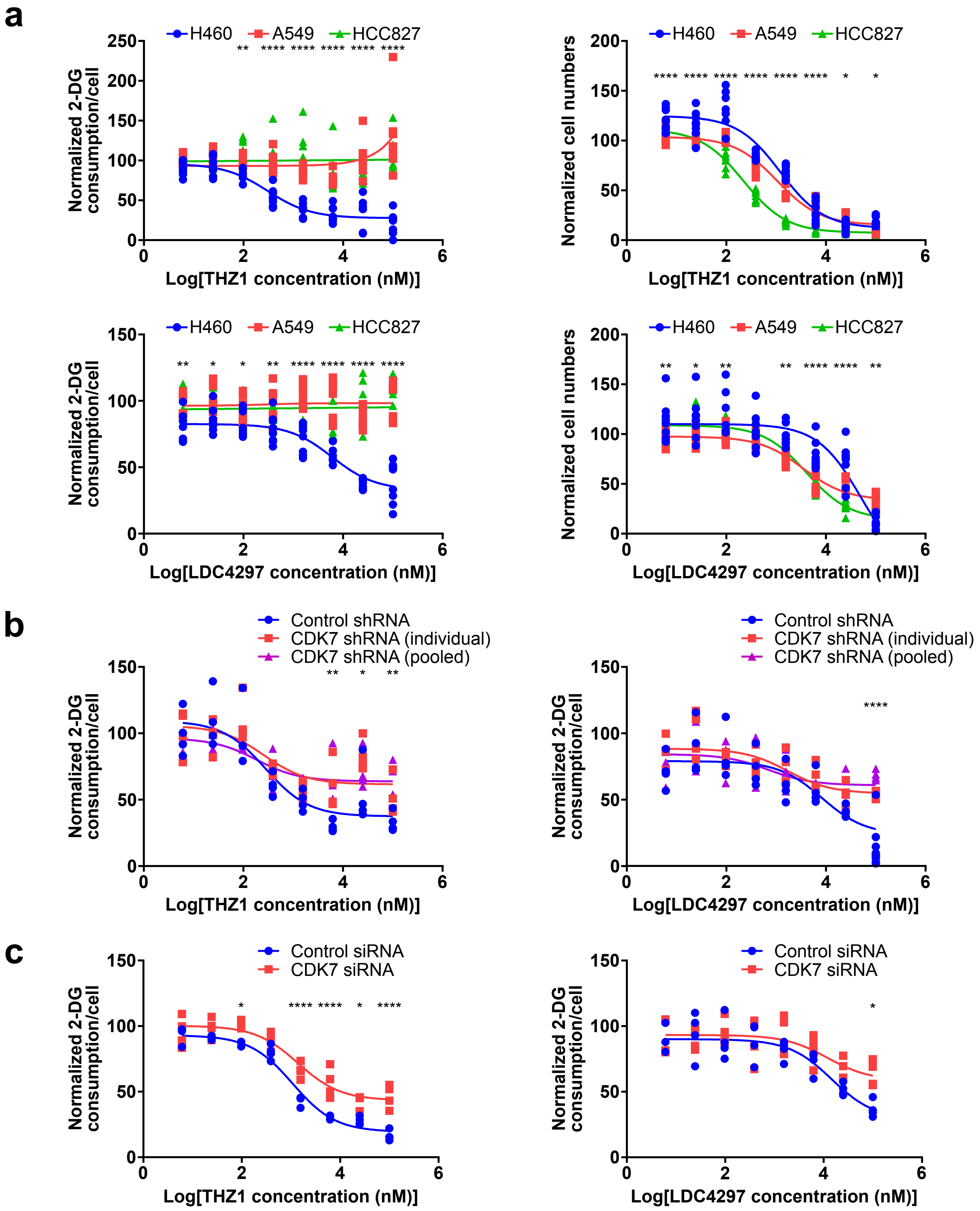

Supplementary Figure 10. THZ1 and LDC4297 inhibit glucose consumption in H460 cells. a, Glucose consumption (*left*) and cell growth (*right*) dose response curves in H460, A549, and HCC827 cells treated with THZ1 (*top*) or LDC4297 (*bottom*). *n*=8. P values determined by two-way ANOVA tests. **b,** Glucose consumption dose response curves in H460 cells transfected with control shRNA or shRNA targeted against CDK7 and treated with THZ1 (*left*) or LDC4297 (*right*). *n*=4. P values determined by two-way ANOVA tests. **c,** Glucose consumption dose response curves in H460 cells transfected with control siRNA or siRNA targeted against CDK7 and treated with THZ1 (*left*) or LDC4297 (*right*). *n*=4. P values determined by two-way ANOVA tests. *: P<0.05; **: P<0.01; ****: P<0.0001.

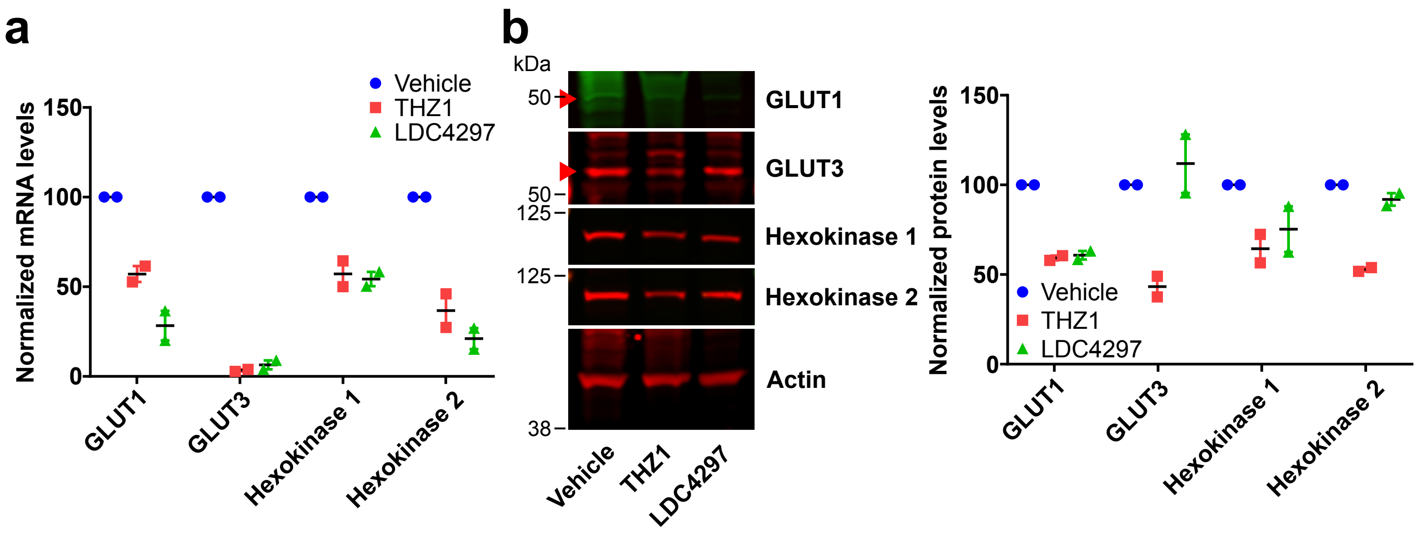

Supplementary Figure 11. THZ1 and LDC4297 inhibit GLUT1 mRNA and protein levels in H460 cells. **a,** mRNA levels from H460 cells treated with vehicle, THZ1 (10 μM), or LDC4297 (10 μM). *n*=2. **b,** Immunoblots (*left*) and quantification (*right*) of lysate from H460 cells treated with vehicle, THZ1 (10 μM), or LDC4297 (10 μM). *n*=2. Data are plotted as mean ± SEM.

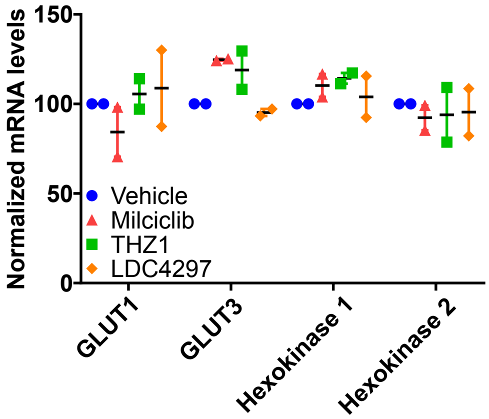

Supplementary Figure 12. Milciclib, THZ1, and LDC4297 do not decrease GLUT1 mRNA levels in HCC827 cells. mRNA levels from HCC827 cells treated with vehicle or Milciclib. *n*=2. Data are plotted as mean ± SEM.

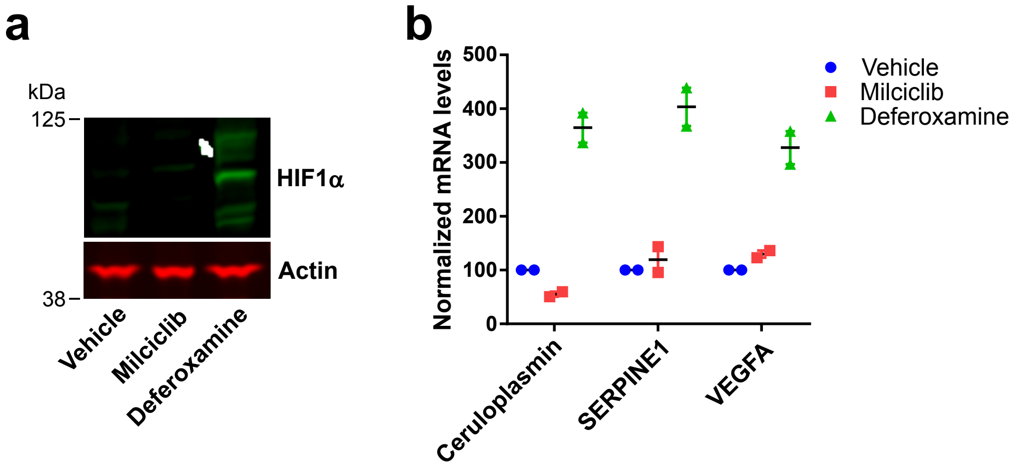

Supplementary Figure 13. Milciclib does not induce HIFα and has no consistent effect on HIFα-regulated gene products. **a,** Immunoblots of lysate from H460 cells treated with vehicle or Milciclib (10 μM) or with Deferoxamine (to induce HIF1α). *n*=2. **b,** mRNA levels from H460 cells treated with vehicle or Milciclib, or Deferoxamine (100 μM). *n*=2. Data are plotted as mean ± SEM.

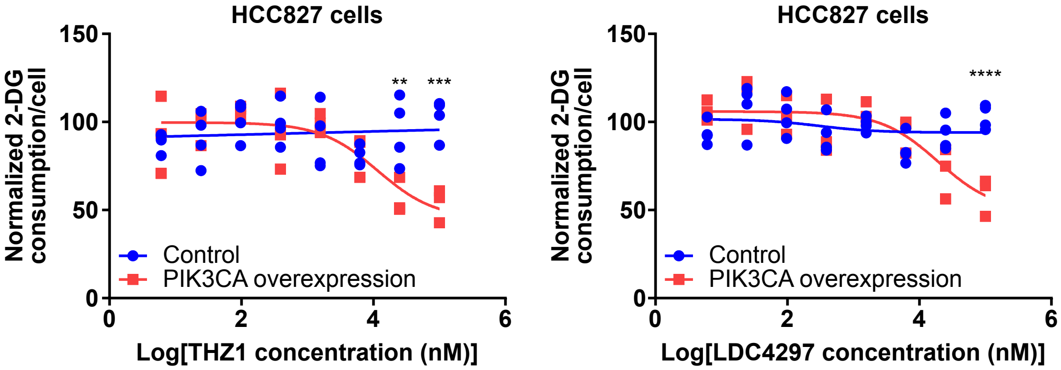

Supplementary Figure 14. PIK3CA overexpression sensitizes HCC827 cells to the glucose inhibitory effect of THZ1 and LDC4297. Glucose consumption dose response curves of HCC827 cells transfected with a control or PIK3CA overexpression plasmid and treated with THZ1 (*left*) or LDC4297 (*right*). Control: n=4, PIK3CA overexpression: n=3 for both compounds. P values determined by two-way ANOVA tests. **: P<0.01; ***: P<0.001; ****: P<0.0001.

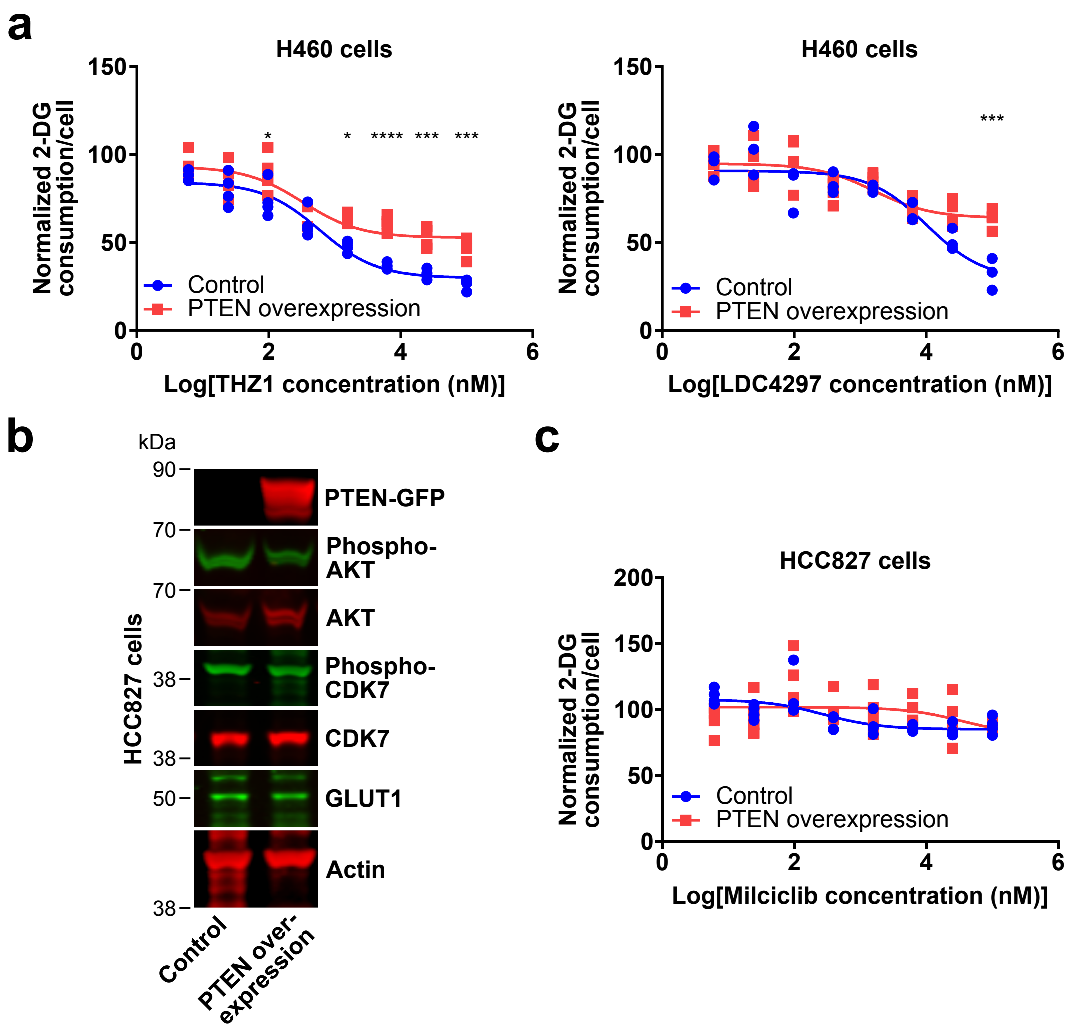

Supplementary Figure 15. PTEN overexpression desensitizes H460 cells to the inhibitory effect of THZ1 and LDC4297 on glucose consumption but has no effect in HCC827 cells. a, Glucose consumption dose response curves of H460 cells transfected with a control or PTEN overexpression plasmid and treated with THZ1 or LDC4297. THZ1: *n*=4; LDC4297: Control: *n*=3, PTEN overexpression: *n*=4. P values determined by two-way ANOVA tests. **b,** Immunoblots of lysate from HCC827 cells transfected with a control or PTEN overexpression plasmid. *n*=2. **c,** Glucose consumption dose response curves of HCC827 cells transfected with a control of PTEN overexpression plasmid and treated with Milciclib. *n*=4. *: P<0.05; ***: P<0.001; ****: P<0.0001.

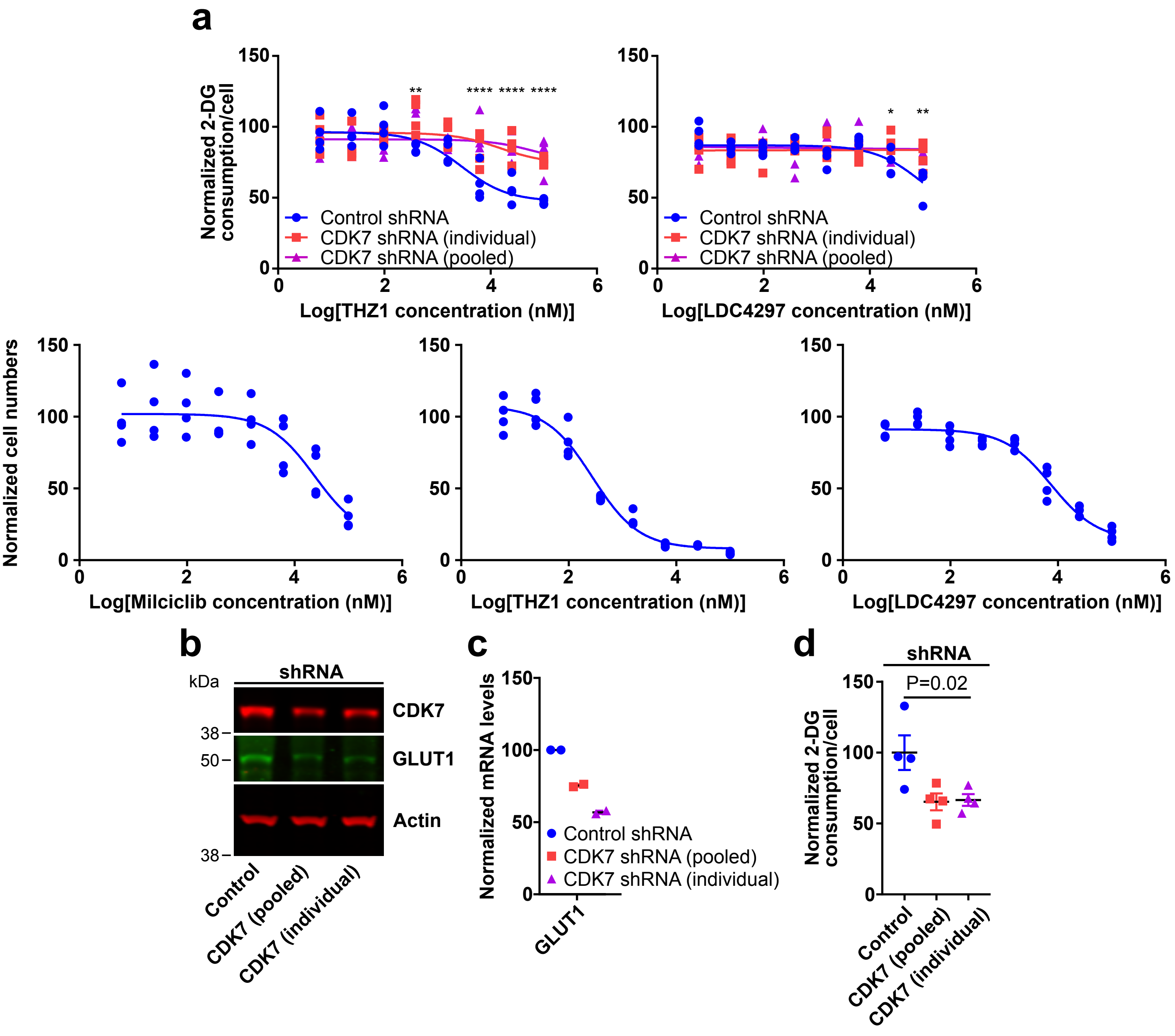

Supplementary Figure 16. Milciclib, THZ1, and LDC4297 block glucose consumption in H1975 cells by inhibiting CDK7. a, Glucose consumption dose response curves in H1975 cells transfected with control shRNA or shRNA targeted against CDK7 and treated with THZ1 or LDC4297 (*top*). Cell growth dose response curves in H1975 cells treated with Milciclib, THZ1, or LDC4297 (*bottom*). *n*=4. P values determined by two-way ANOVA tests. **b,** Immunoblots of lysate from H1975 cells transfected with control shRNA or shRNA targeted against CDK7. *n*=2. **c,** mRNA levels from H1975 cells transfected with control shRNA or pooled or individual shRNA targeted against CDK7. *n*=2. **d,** Normalized glucose consumption in H1975 cells transfected with control shRNA or shRNA targeted against CDK7. *n*=4. P value determined by a one-way ANOVA test. *: P<0.05; **: P<0.01; ****: P<0.0001. Data are plotted as mean ± SEM.

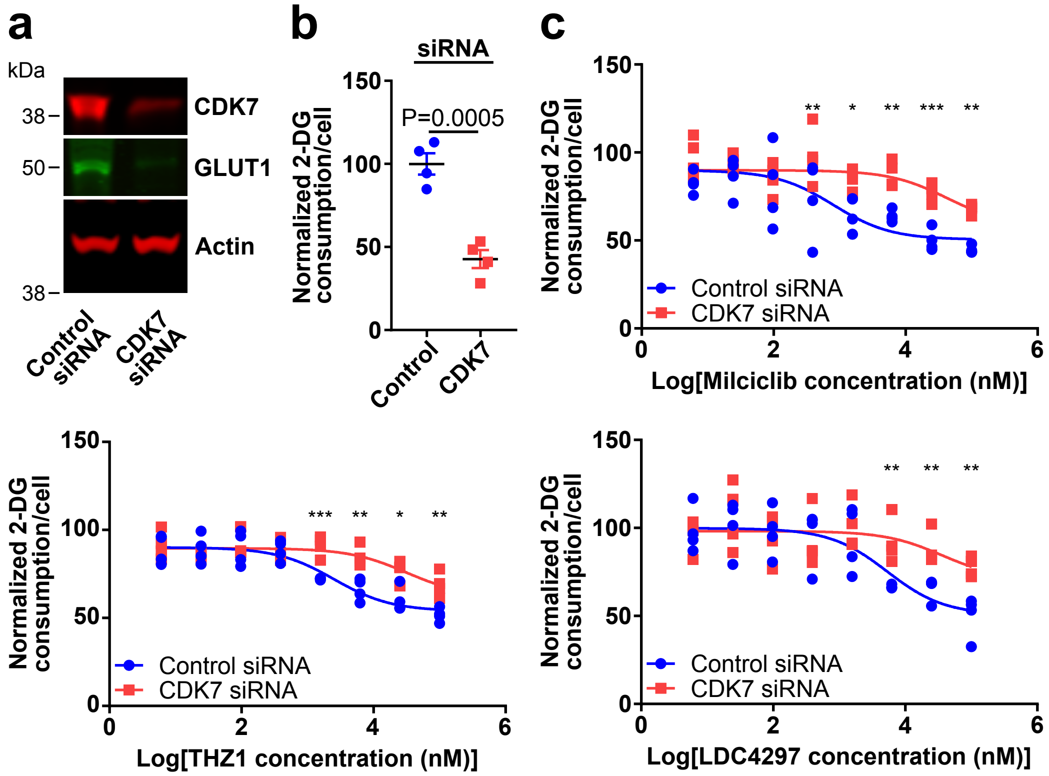

Supplementary Figure 17. CDK7 promotes glucose consumption in H1975 cells. **a,** Immunoblots of lysate from H1975 cells transfected with control siRNA or siRNA targeted against CDK7. *n*=2. **b,** Glucose consumption in H1975 cells transfected with control siRNA or siRNA targeted against CDK7. *n*=4. P value determined by an unpaired t test. **c,** Glucose consumption dose response curves in H1975 cells transfected with control siRNA or siRNA targeted against CDK7 and treated with Milciclib, THZ1, or LDC4297. *n*=4. P values determined by two-way ANOVA tests. *: P<0.05; **: P<0.01; ***: P<0.001. Data are plotted as mean ± SEM.

a

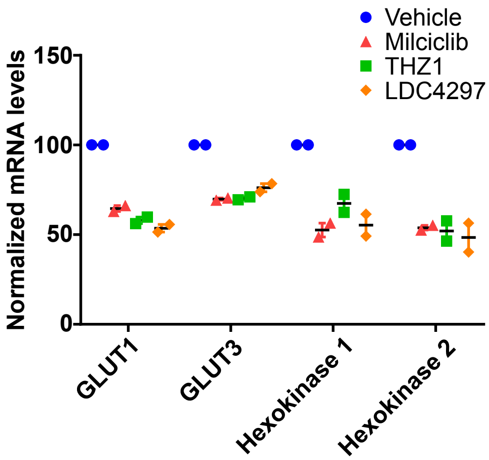

b

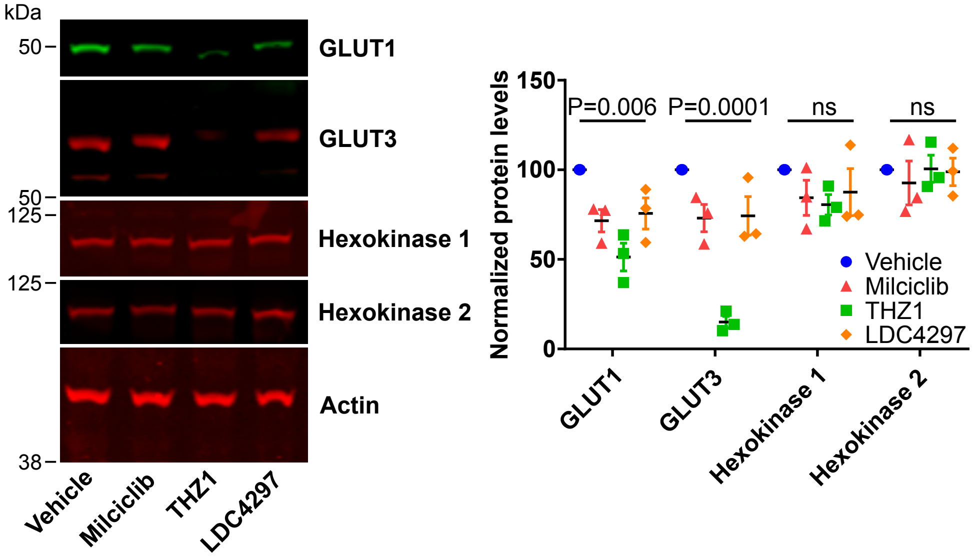

Supplementary Figure 18. Milciclib, THZ1, and LDC4297 decrease GLUT1 mRNA and protein levels in H1975 cells. a, mRNA levels from H1975 cells treated with vehicle or Milciclib (10 μM), THZ1 (10 μM), and LDC4297 (10 μM). *n*=2. **b,** Immunoblots (*left*) and quantification (*right*) of lysate from H1975 cells treated with vehicle, Milciclib (10 μM), THZ1 (10 μM), or LDC4297 (10 μM). *n*=3. P values determined by one-way ANOVA tests. ns: not significant. Data are plotted as mean ± SEM.

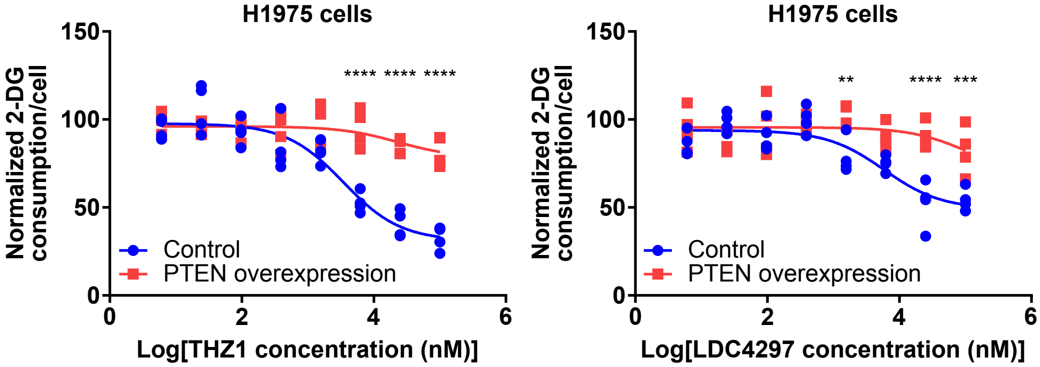

Supplementary Figure 19. PTEN overexpression desensitizes H1975 cells to the inhibitory effects of THZ1 and LDC4297 on glucose consumption. Glucose consumption dose response curves of H1975 cells transfected with a control or PTEN overexpression plasmid and treated with THZ1 and LDC4297. *n*=4. P values determined by two-way ANOVA tests. **: P<0.01; ***: P<0.001; ****: P<0.0001.

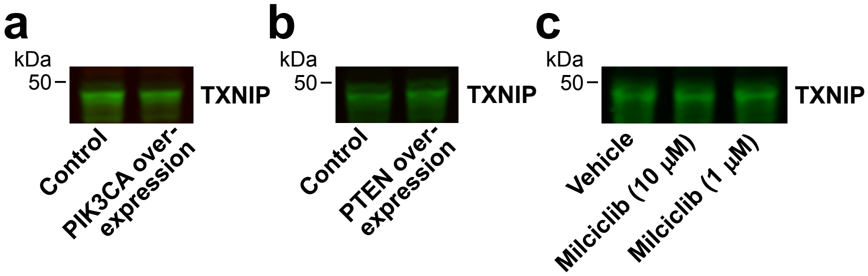

Supplementary Figure 20. TXNIP levels are not affected by PIK3CA or PTEN overexpression or by Milciclib treatment. **a,** Immunoblot of lysate from HCC827 cells transfected with a control or PIK3CA overexpression plasmid. TXNIP levels in the PIK3CA overexpression cells are 97±8.3% of TXNIP levels in the control cells. Corresponding actin immunoblot for these samples is displayed in **Figure 6a**. *n*=2. **b,** Immunoblot of lysate from H460 cells transfected with a control or PTEN overexpression plasmid. TXNIP levels in the PTEN overexpression cells are 116±6.5% of TXNIP levels in the control cells. Corresponding actin immunoblot for these samples is displayed in **Figure 6c**. *n*=2. **c,** Immunoblot of lysate from H460 cells treated with vehicle or Milciclib. TXNIP levels in the cells treated with 10 μM Milciclib are 82±8.4% of TXNIP levels in the vehicle-treated cells. Corresponding actin immunoblot for these samples is displayed in **Supplementary Figure 5**. *n*=2. Data are presented as mean ± SEM.

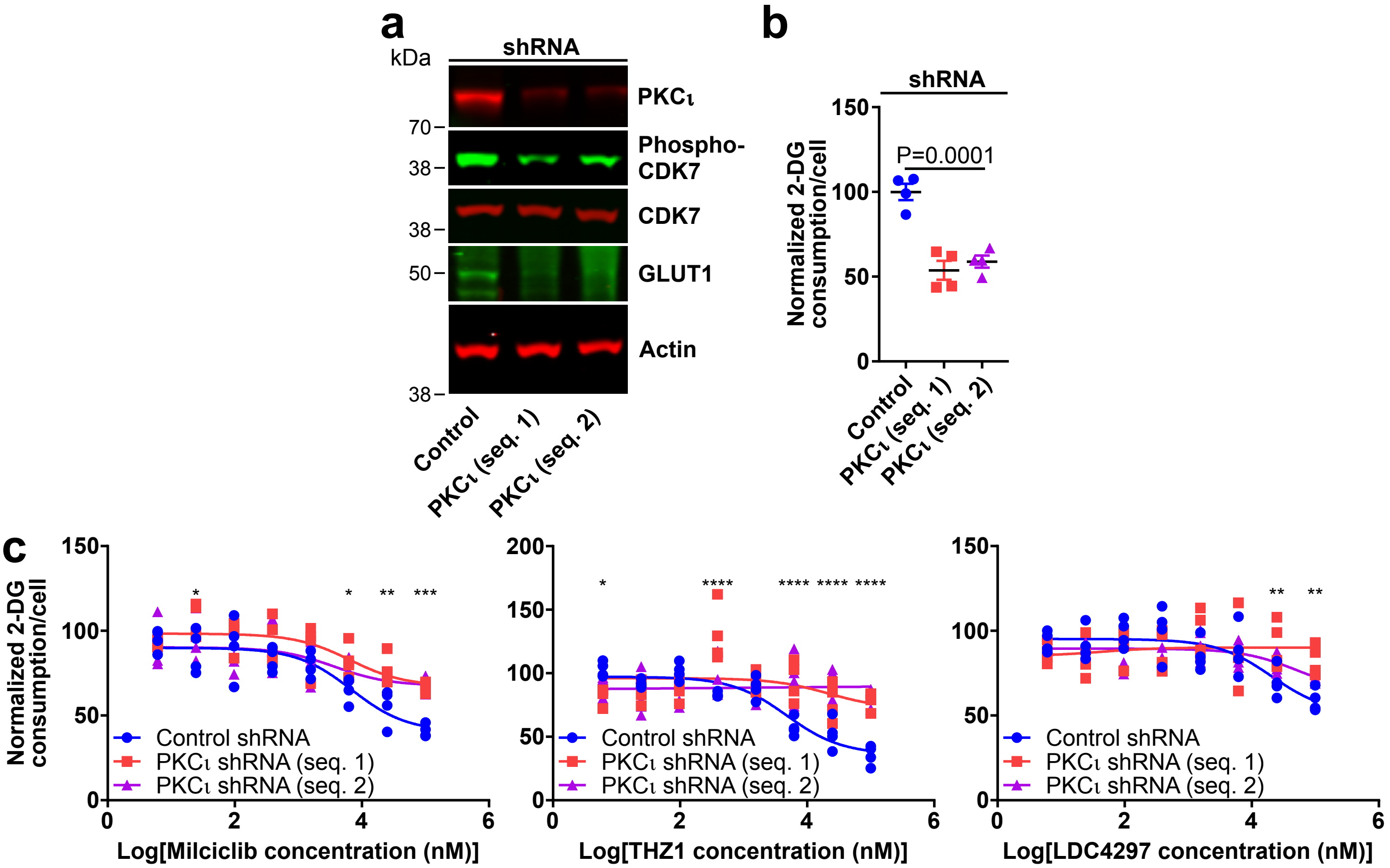

Supplementary Figure 21. Milciclib, THZ1, and LDC4297 block glucose consumption in H1975 cells downstream of PKCι. a, Immunoblots of lysate from H1975 cells transfected with control shRNA or shRNA targeted against PKCι. *n*=2. **b,** Glucose consumption in H1975 cells transfected with control shRNA or shRNA targeted against PKCι. *n*=4. P value determined by a one-way ANOVA test. **c,** Glucose consumption dose response curves in H1975 cells transfected with control shRNA or shRNA targeted against PKCι and treated with Milciclib, THZ1, or LDC4297. *n*=4. P values determined by two-way ANOVA tests. *: P<0.05; **: P<0.01; ***: P<0.001; ****: P<0.0001. Data are plotted as mean ± SEM.

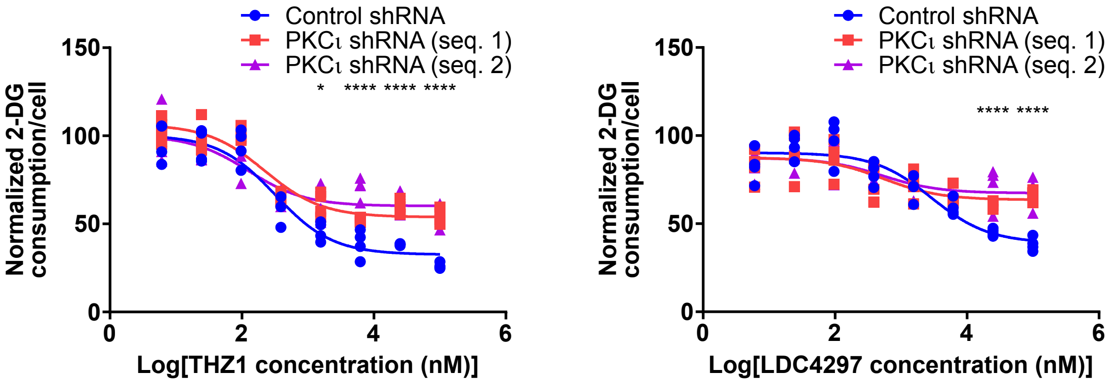

Supplementary Figure 22. PKCι knockdown blocks the inhibition of glucose consumption by THZ1 and LDC4297 in H460 cells. Glucose consumption dose response curves in H460 cells transfected with control shRNA or shRNA targeted against PKCι and treated with THZ1 (*left*) or LDC4297 (*right*). Seq. 1 and Seq. 2 represent different shRNA sequences. *n*=4. P values determined by two-way ANOVA tests. *: $P<0.05$; ****: $P<0.0001$.

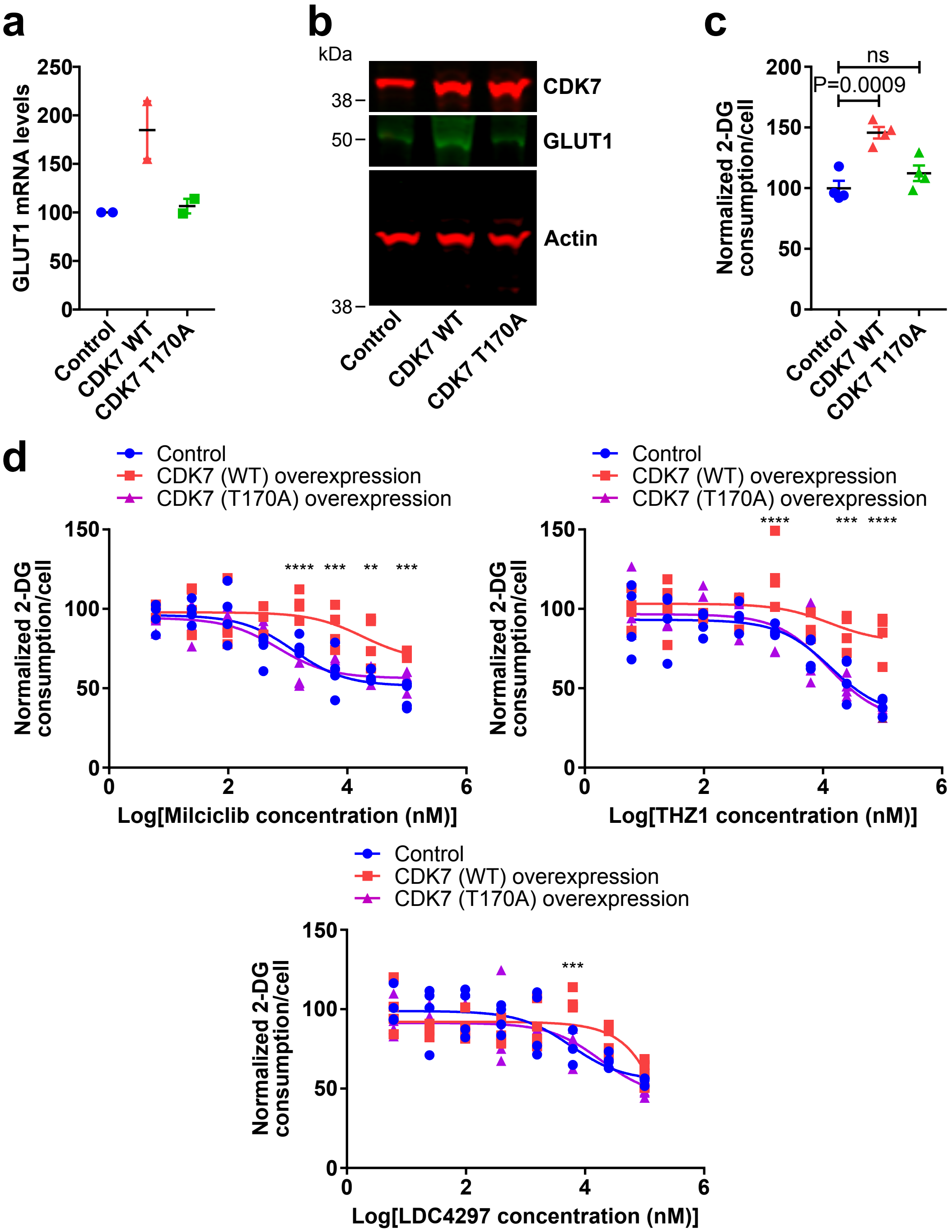

Supplementary Figure 23. Overexpression of wild-type (WT) CDK7 but not phospho-site mutant T170A CDK7 desensitizes H1975 cells to the inhibitory effect of CDK7 inhibitors. a, GLUT1 mRNA levels from H1975 cells transfected with a control, WT CDK7, or T170A mutant CDK7 overexpression plasmid. *n*=2. **b,** Immunoblots of lysate from H1975 cells transfected with a control, WT CDK7, or T170A mutant CDK7 overexpression plasmid. *n*=2. **c,** Glucose consumption in H1975 cells transfected with a control, WT CDK7, or T170A mutant CDK7 overexpression plasmid. *n*=4. P values determined by a one-way ANOVA test. **d,** Glucose consumption dose response curves in H1975 cells transfected with a control, WT CDK7, or T170A mutant CDK7 overexpression plasmid and treated with Milciclib, THZ1, and LDC4297. *n*=4. P values determined by two-way ANOVA tests. **: P<0.01; ***: P<0.001; ****: P<0.0001. Data are plotted as mean ± SEM.

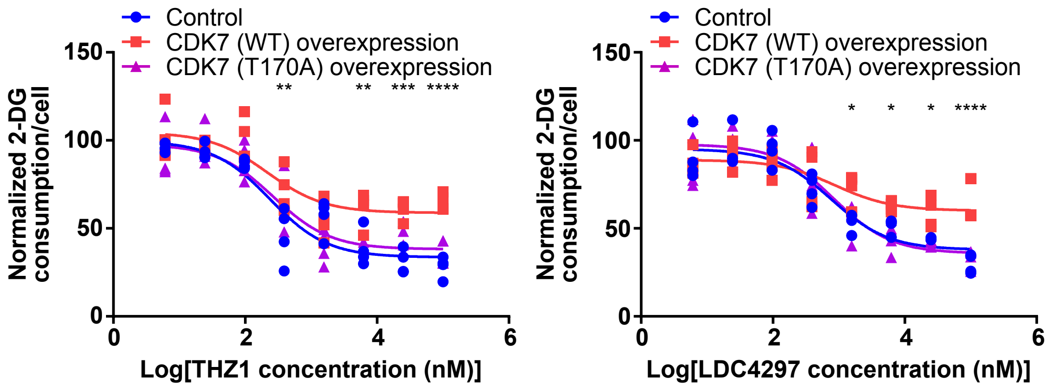

Supplementary Figure 24. Overexpression of wild-type (WT) CDK7 but not phospho-site mutant T170A CDK7 desensitizes H460 cells to the inhibitory effects of THZ1 and LDC4297 on glucose consumption. Glucose consumption dose response curves in H460 cells transfected with a control, WT CDK7, or T170A mutant CDK7 overexpression vector and treated with THZ1 or LDC4297. *n*=4. *: P<0.05; **: P<0.01; ***: P<0.001; ****: P<0.0001. P values determined by two-way ANOVA tests.

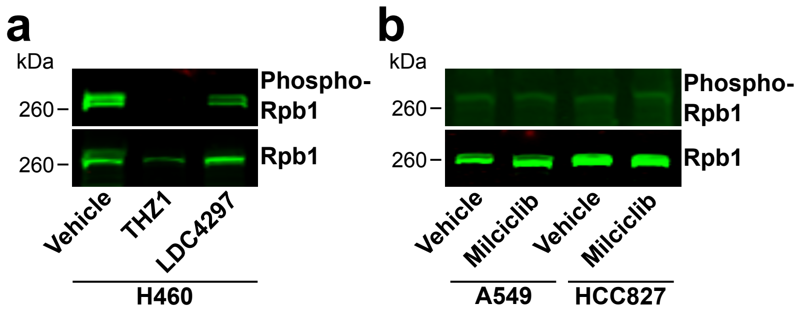

Supplementary Figure 25. THZ1 and LDC4297 decrease phospho-Rpb1 levels in H460 cells. a, Immunoblots of cell lysate from H460 cells treated with vehicle, THZ1 (10 μM), or LDC4297 (10 μM). *n*=2. Corresponding actin immunoblot for these samples is displayed in **Supplementary Figure 11**. **b,** Immunoblots of cell lysate from A549 and HCC827 cells treated with vehicle or Milciclib (10 μM). Corresponding actin immunoblot for these samples is displayed in **Supplementary Figure 6**. *n*=2.

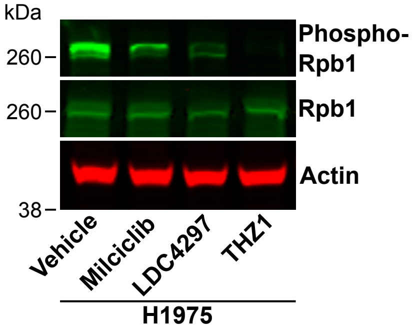

Supplementary Figure 26. Milciclib, LDC4297, and THZ1 decrease phospho-Rpb1 levels in H1975 cells. Immunoblots of cell lysate from H1975 cells treated with vehicle, Milciclib (10 μM), LDC4297 (10 μM), and THZ1 (10 μM). *n*=2.

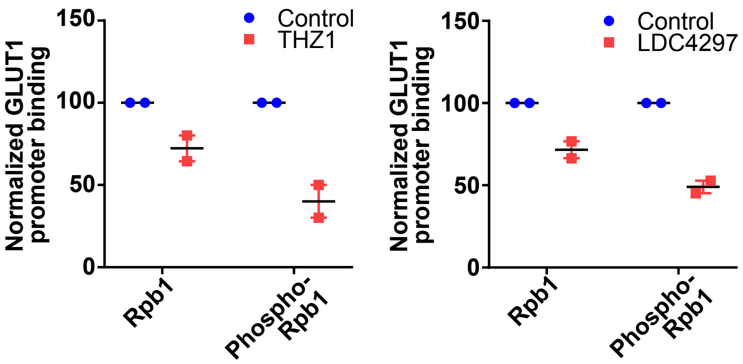

Supplementary Figure 27. THZ1 and LDC4297 decrease phospho-Rpb1 levels on the GLUT1 promoter in H460 cells. Normalized Rpb1 and phospho-Rpb1 levels on the GLUT1 promoter in H460 cells treated with vehicle, THZ1 (10 μM), or LDC4297 (10 μM), as determined by chromatin immunoprecipitation and quantitative PCR. *n*=2. Data are plotted as mean ± SEM.

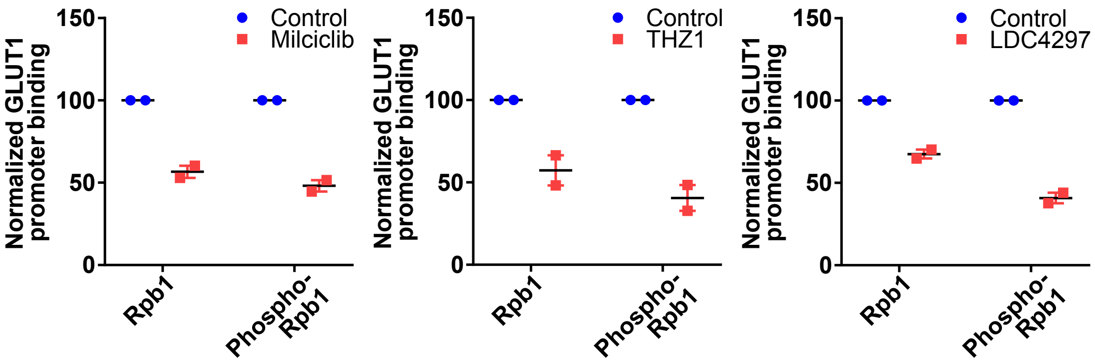

Supplementary Figure 28. Milciclib, THZ1, and LDC4297 decrease phospho-Rpb1 levels on the GLUT1 promoter in H1975 cells. Normalized Rpb1 and phospho-Rpb1 levels on the GLUT1 promoter in H1975 cells treated with vehicle, Milciclib (10 μM), THZ1 (10 μM), or LDC4297 (10 μM), as determined by chromatin immunoprecipitation and quantitative PCR. *n*=2. Data are plotted as mean ± SEM.

Figure 4b

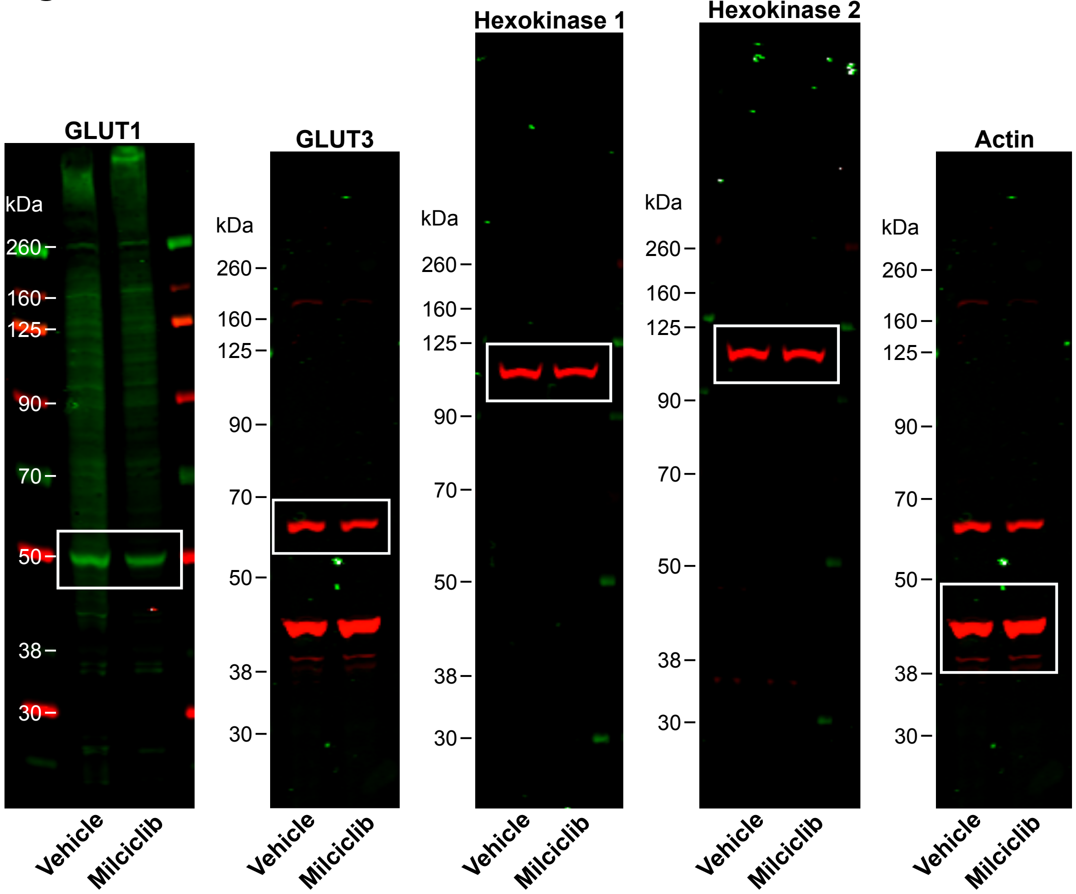

Figure 4d

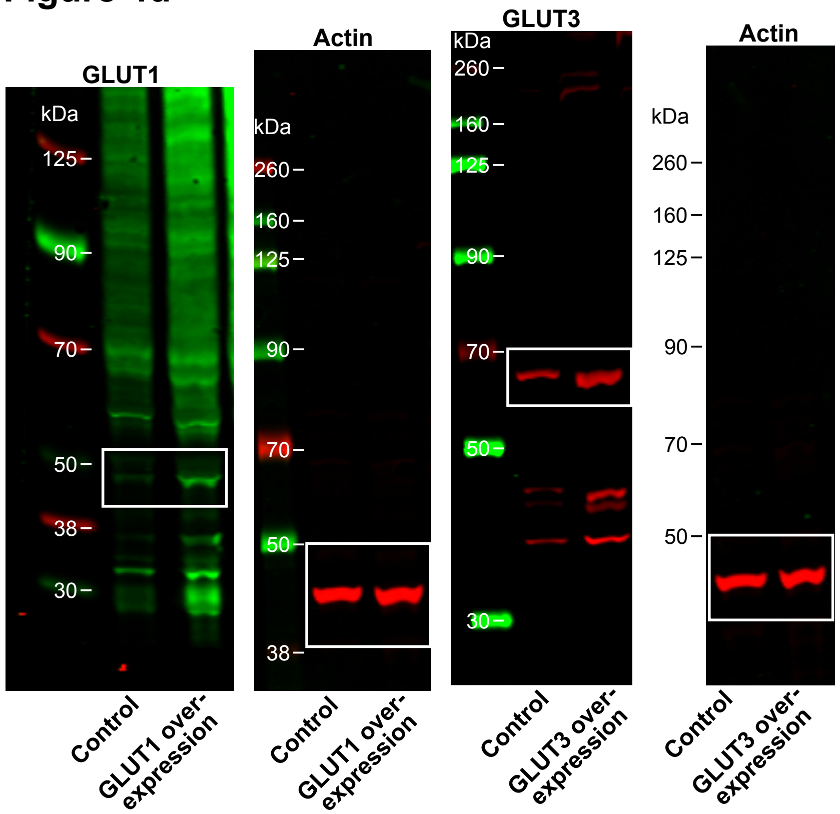

(continued on the next page)

Figure 5a

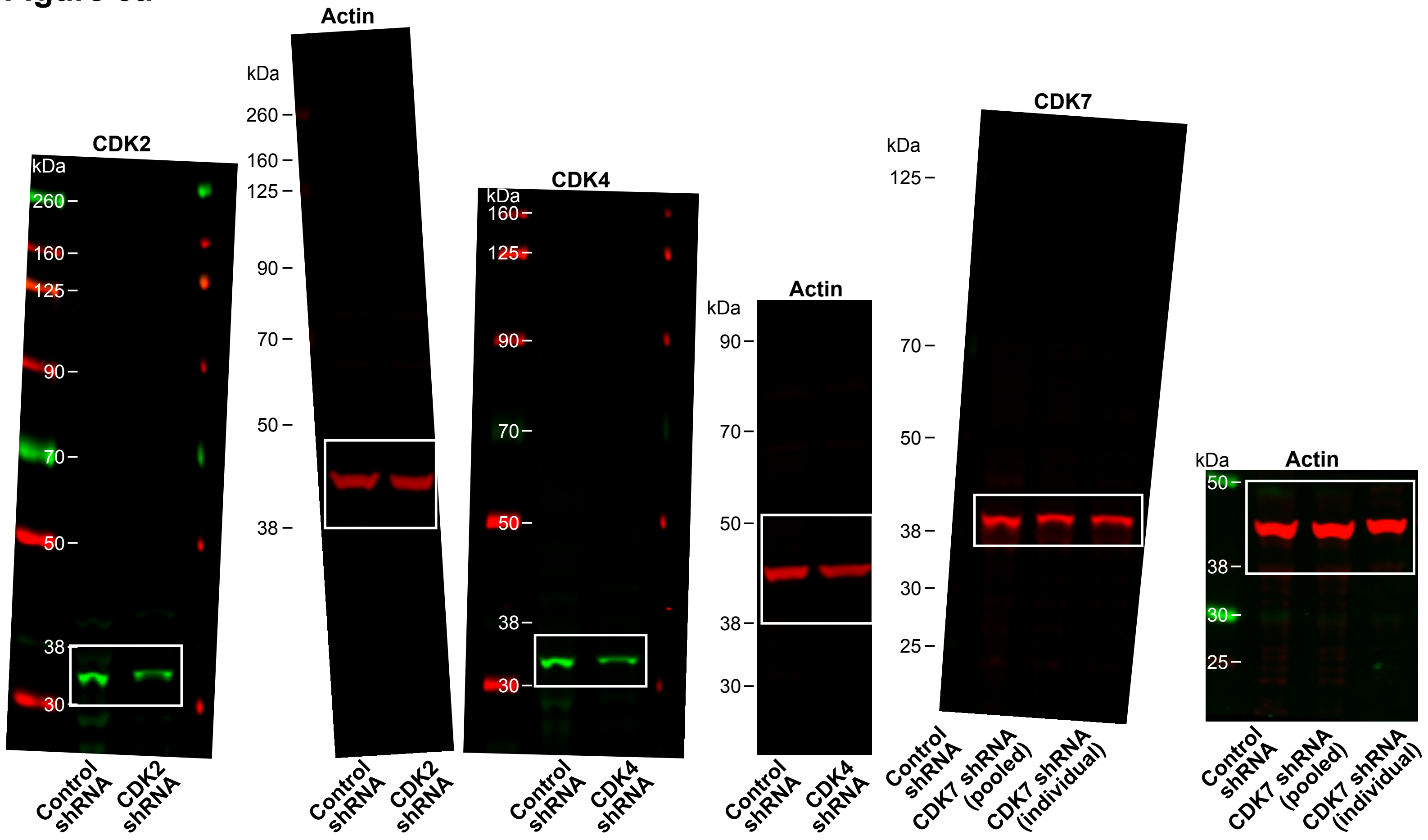

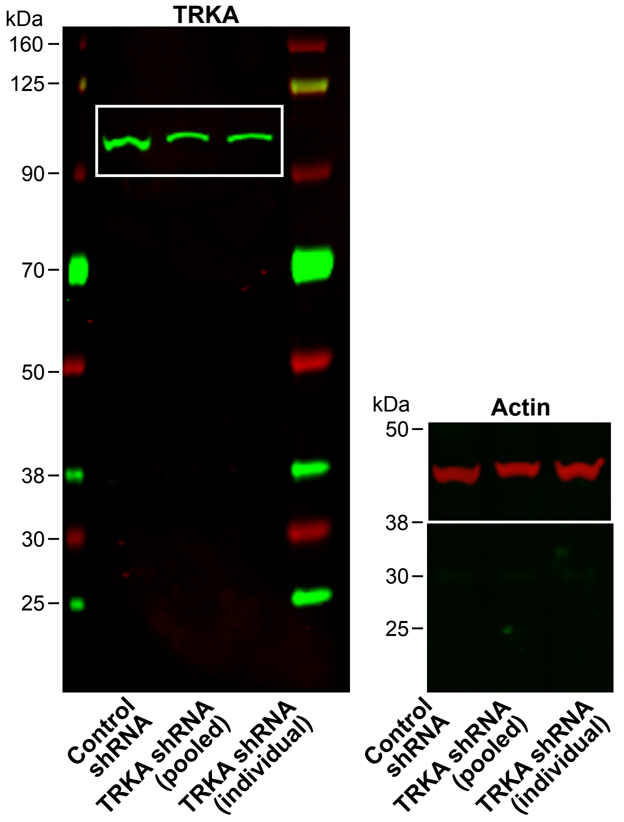

Figure 5f

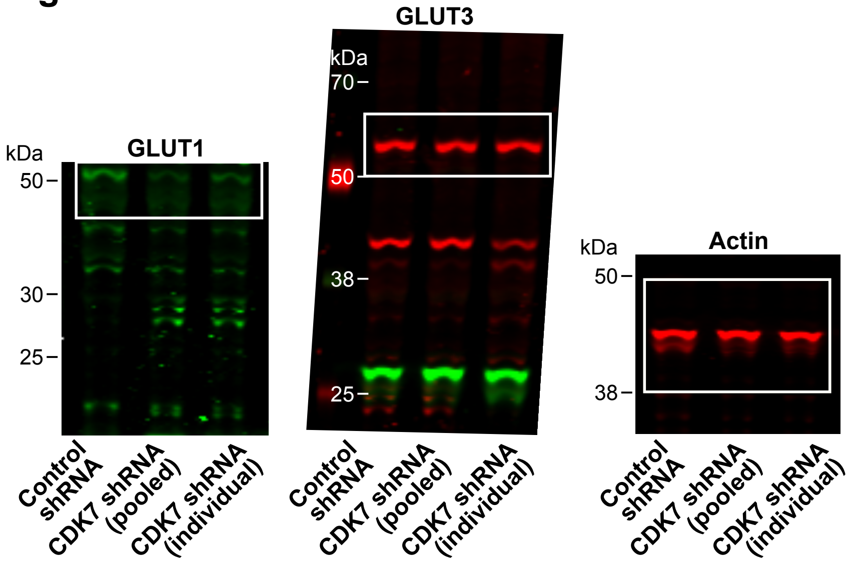

(continued on the next page)

Figure 6a

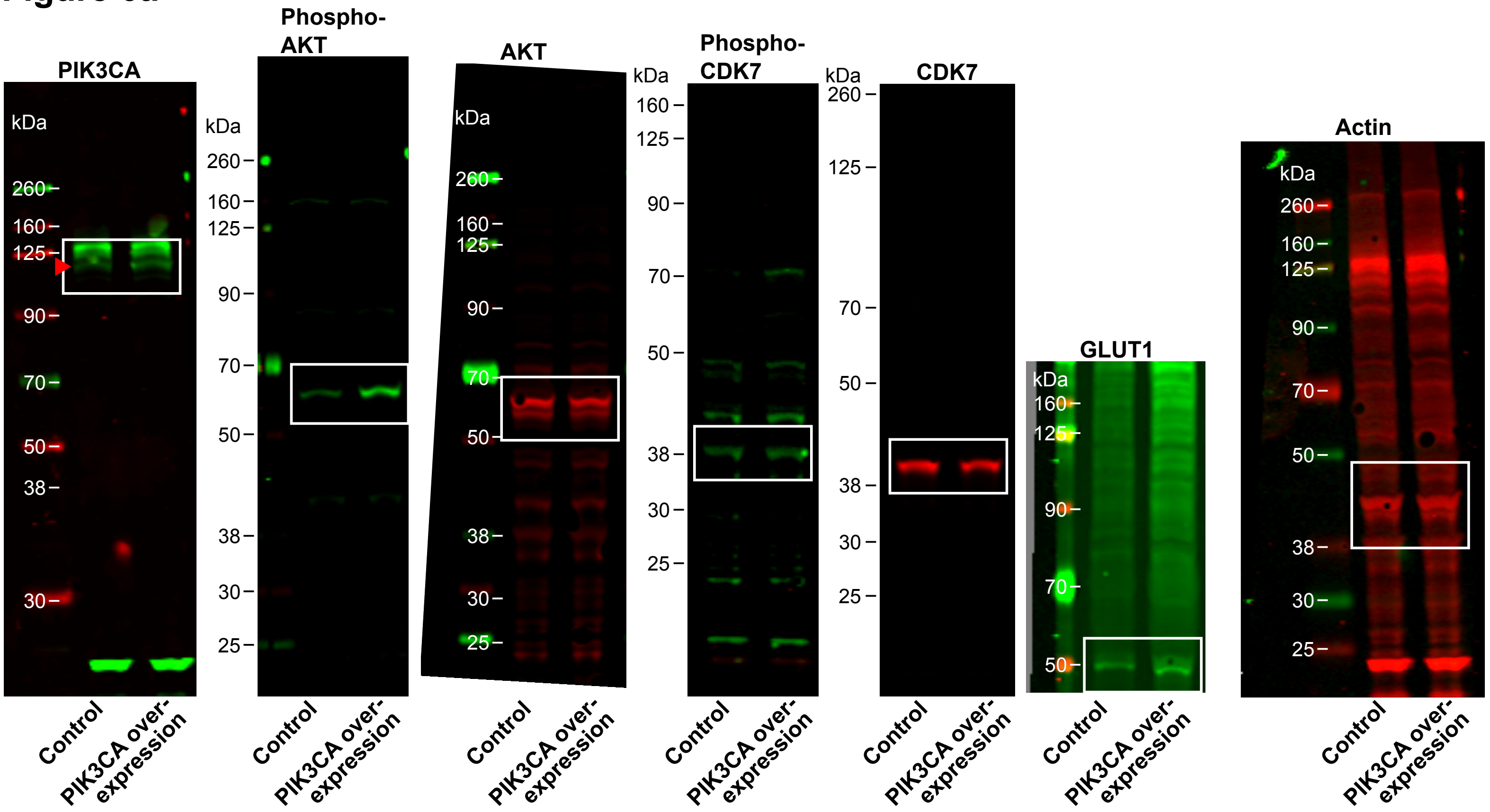

Figure 6c

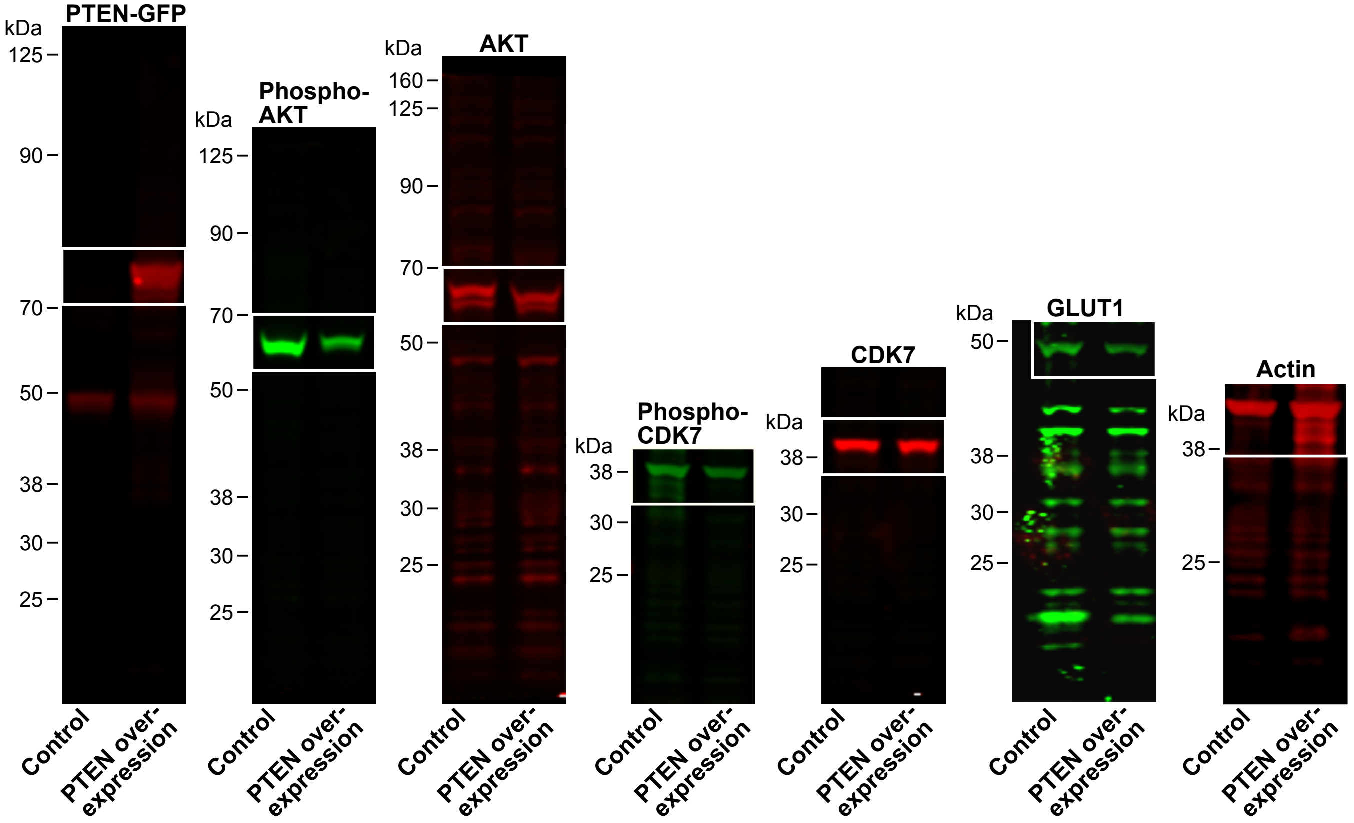

(continued on the next page)

Figure 6f

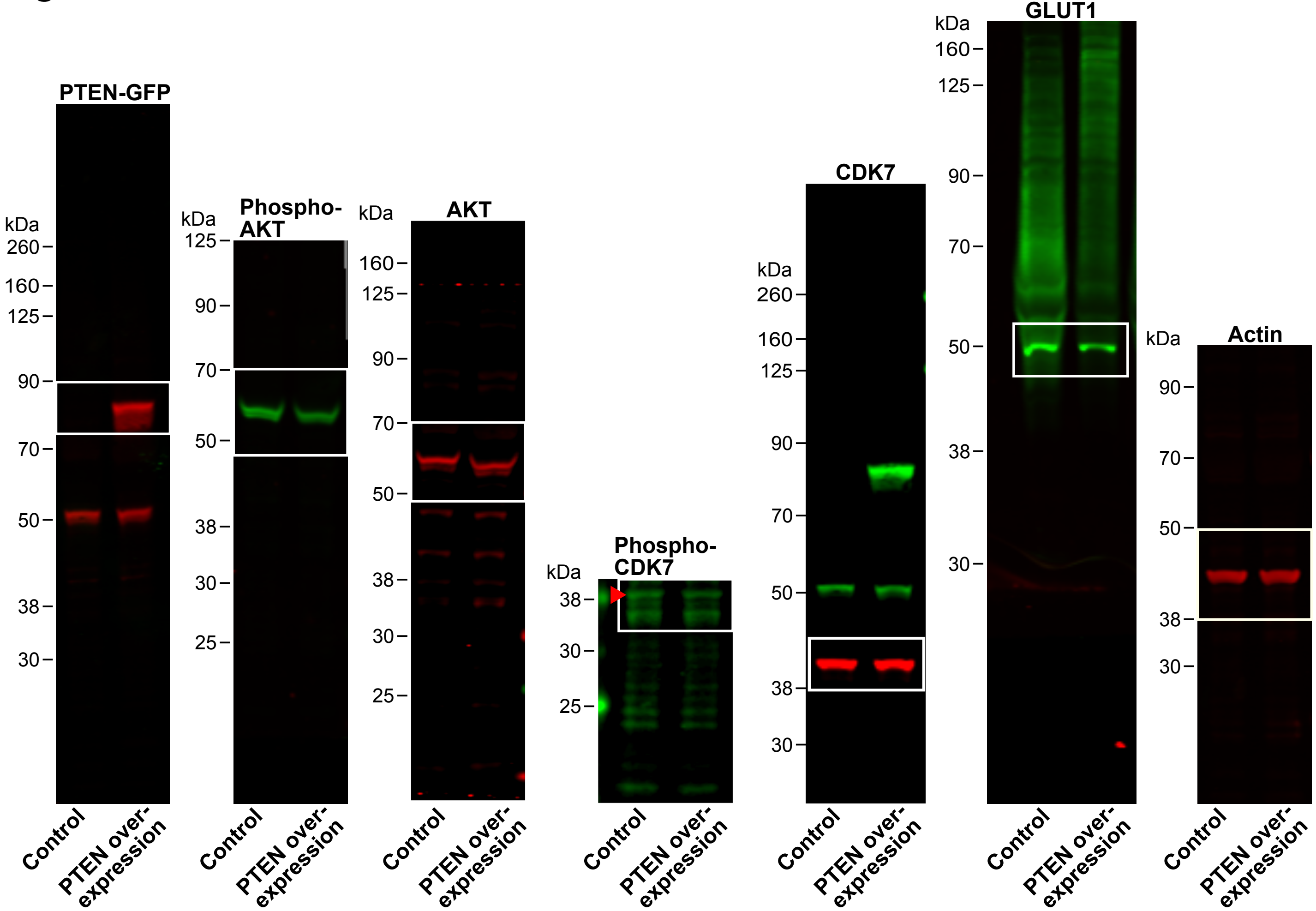

Figure 7a

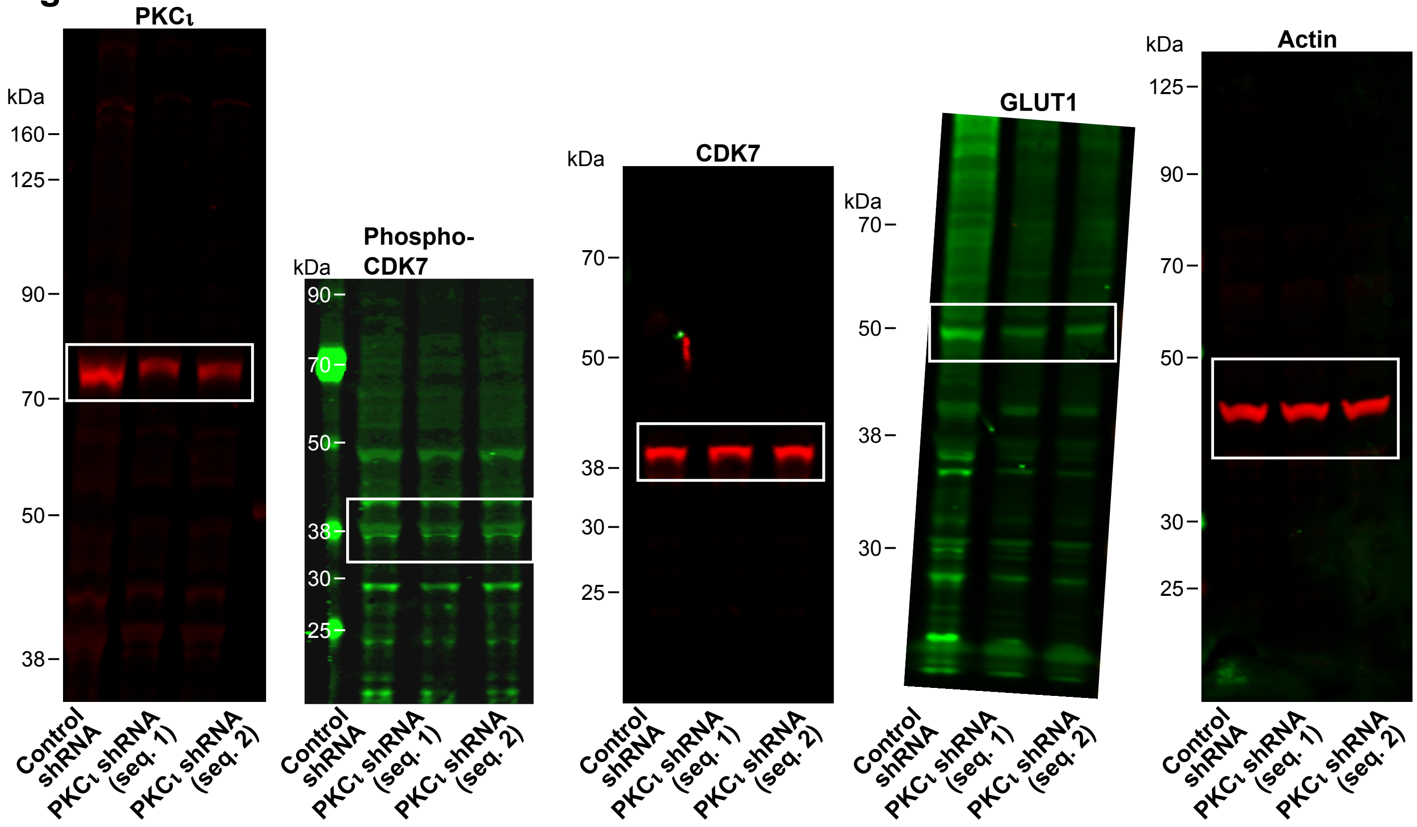

(continued on the next page)

Figure 7e

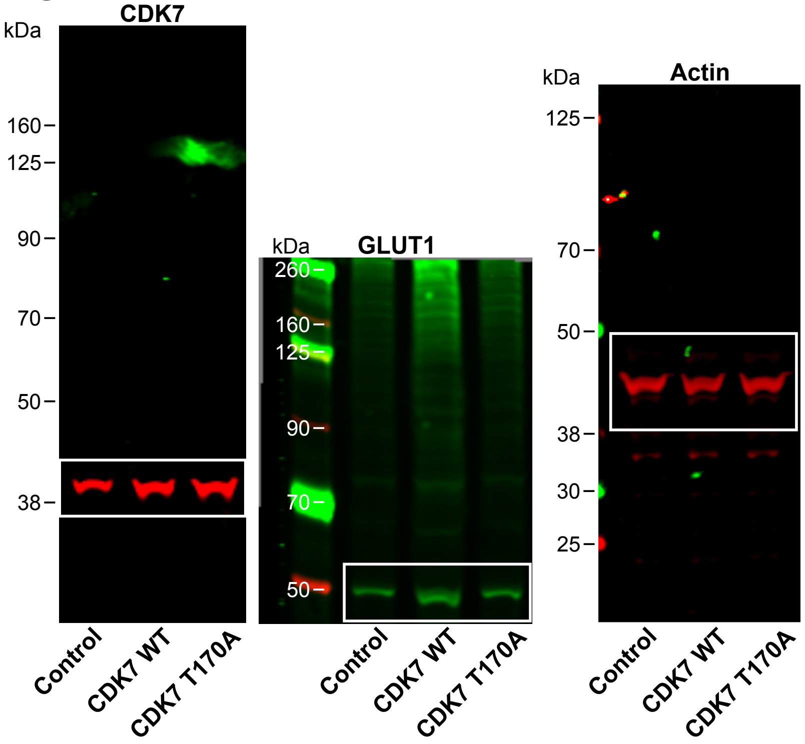

Figure 7h

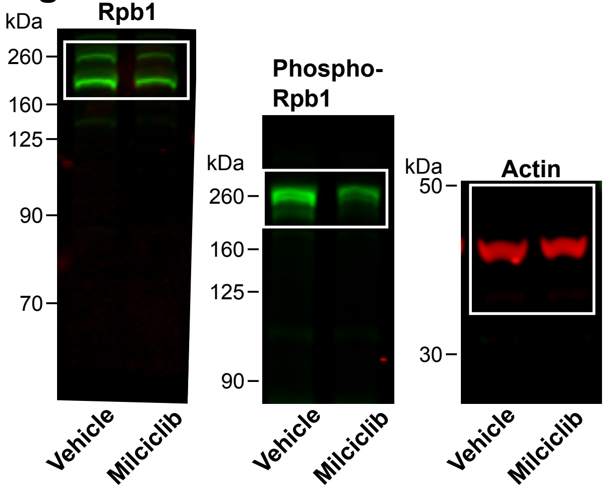

Supplementary Figure 29. Uncropped immunoblots from the main figures.

Supplementary Table 1. Validated small molecule inhibitors of glucose consumption.

A549 cells	H460 cells		HCC827 cells
BMS-536924	5619779	MK-2206 2HCl	AC-93253 iodide
Entrectinib	A-674563	MS-1502111	AEE788
Naringin hydrate	α-Santonin	Niclosamide	AP26113
PD173074	Auranofin	Nintedanib	Apomorphine
PP121	AZ20	Nitidine chloride	AZD3463
TCS 359	AZD2014	Pacritinib	AZD9291
WAY-600	AZD3463	PD-166285	Bay 11-7085
YM201636	Bay 11-7085	PD-173074	β-Lapachone
	β-Lapachone	PD-180970	Bosutinib
	BGT226	PF-00562271	Camptothecin (S,+)
	BMS-536924	PF-04691502	CEP-32496
	Brefeldin A	PF-431396	CGK 733
	Camptothecin (S,+)	PI-103	CHIR-124
	Crenolanib	Ponatinib	Crenolanib
	CUDC-101	SAM001246648	CX-6258 HCl
	CUDC-907	SAM001247018	Dovitinib
	Dipyridamole	Sanguinarine chloride	ERK5-IN-1
	Dorsomorphin 2HCl	TAK-901	GDC-0980
	Dovitinib	TCS 359	Gefitinib
	Ellipticine	TG101209	GF109203X
	Entrectinib	Tie2 kinase inhibitor	GSK-3 Inhibitor IX
	GDC-0941	Topotecan HCl	GZD824
	GSK1838705A	Tyrphostin 9	Lapatinib
	GSK2126458	Tyrphostin AG 879	Naloxone HCl
	GSK2334470	URMC-099	Nitidine chloride
	IMD 0354	VE-821	NSC 95397
	K114	VE-822	PD-173955
	Lapatinib	Volasertib	PD-180970
	LY2835219	WZ3146	PP2
	Milciclib	ZSTK474	PP242
	Mitoxantrone 2HCl		R(-)-Propylnorapo-morphine HCl
			Ro3280
			SGI-1776
			Sotrastaurin
			TAE226
			Tivozanib
			VE-822
			VS-5584
			WHI-P154
			WZ3146
			WZ4003
			WZ8040
			YM201636
			ZM 323881 HCl

Supplementary Table 2. Small molecule screening data

Category	Parameter	Description
Assay	Type of assay	Cell-based
	Target	Glucose consumption
	Primary measurement	Detection of 2-DG-6-phosphate in cells treated with 2-DG
	Key reagents	Please see the "High-throughput 2-DG luminescence assay" section of the Methods for reagents used in this assay
	Assay protocol	Please see the "High-throughput 2-DG luminescence assay" section of the Methods for the assay protocol
	Additional comments	None
Library	Library size	3555 compounds
	Library composition	Known bioactives
	Source	The Selleck Chemicals kinase inhibitor library, the Prestwick FDA-approved drug library, the LOPAC collection, and the NIH clinical collection small molecule libraries
	Additional comments	None
Screen	Format	384 well plate
	Concentration(s) tested	10 μM
	Plate controls	Positive control: Cytochalasin B<br>Negative control: DMSO
	Reagent/ compound dispensing system	Reagents were added with a Multidrop 384 manifold liquid dispenser or a BioTek EL406 Microplate washer/dispenser. Plates were washed with a BioTek EL406 Microplate washer/dispenser.
	Detection instrument and software	EnVision Multilabel Plate Reader using the EnVision software
	Assay validation/QC	Z factor
	Correction factors	None
	Normalization	For each well, luminescence was normalized to total cell numbers (determined from the fluorescent microscope image), and values for individual wells were normalized to the DMSO control for that row.
	Additional comments	None
Post-HTS analysis	Hit criteria	>50% decrease in glucose consumption
	Hit rate	Hit rate of small molecules identified in the primary screen: 1.8%; Hit rate of validated small molecules: 1.1%
	Additional assay(s)	Hits from the primary screen were rescreened using the same assay to identified validated hits. A subset of the validated hits were further analyzed by identifying glucose consumption IC_{50} values. Milciclib was analyzed using ^{3}H-2-DG consumption, media glucose consumption, and *in vivo* ^{18}F-FDG assays.
	Confirmation of hit purity and structure	Compounds were repurchased from Selleck Chem, Cayman Chemical, and Sigma Aldrich
	Additional comments	None
