# Supplementary material for: A high-throughput screen identifies that CDK7 activates glucose consumption in lung cancer cells
Source: Nat Commun. 2019 Nov 29;10:5444. doi: 10.1038/s41467-019-13334-8 (PMC6884612; doi:10.1038/s41467-019-13334-8)
Supplement: Supplementary file 2 — Reporting Summary [file 41467_2019_13334_MOESM2_ESM.pdf]

nature research

Corresponding author(s): Peter M. Clark

Last updated by author(s): Oct 29, 2019

Reporting Summary

Nature Research wishes to improve the reproducibility of the work that we publish. This form provides structure for consistency and transparency in reporting. For further information on Nature Research policies, seeAuthors & Referees and theEditorial Policy Checklist .

Statistics

For all statistical analyses, confirm that the following items are present in the figure legend, table legend, main text, or Methods section.

n/a	Confirmed	
☐	☒	The exact sample size (*n*) for each experimental group/condition, given as a discrete number and unit of measurement
☐	☒	A statement on whether measurements were taken from distinct samples or whether the same sample was measured repeatedly
☐	☒	The statistical test(s) used AND whether they are one- or two-sided<br>*Only common tests should be described solely by name; describe more complex techniques in the Methods section.*
☒	☐	A description of all covariates tested
☐	☒	A description of any assumptions or corrections, such as tests of normality and adjustment for multiple comparisons
☐	☒	A full description of the statistical parameters including central tendency (e.g. means) or other basic estimates (e.g. regression coefficient) AND variation (e.g. standard deviation) or associated estimates of uncertainty (e.g. confidence intervals)
☐	☒	For null hypothesis testing, the test statistic (e.g. F, t, r) with confidence intervals, effect sizes, degrees of freedom and P value noted<br>*Give P values as exact values whenever suitable.*
☒	☐	For Bayesian analysis, information on the choice of priors and Markov chain Monte Carlo settings
☒	☐	For hierarchical and complex designs, identification of the appropriate level for tests and full reporting of outcomes
☒	☐	Estimates of effect sizes (e.g. Cohen's d, Pearson's r), indicating how they were calculated

Our web collection on statistics for biologists contains articles on many of the points above.

Software and code

Policy information about availability of computer code

Data collection	Protein concentrations for Western blots were measured using i-control 1.10 software (Tecan, Mannedorf, CH). Western blot images were acquired using Image Studio 3.1 software (Li-Cor Biosciences, Lincoln, NE). Cells were counted using MetaExpress 6.03.1658 software (Molecular Devices, San Jose, CA). Glucose consumption was measured using Envision Manager 1.13.3009.1409 software (Perkin Elmer, Waltham, MA). microPET images were obtained using G8 PET/CT 2.1.1.0 software (UCLA, Los Angeles, CA) and analyzed using AMIDE 1.0.4 software (Andreas Loening). FRET images were obtained using the MetaFluor Imaging 6.1 software (Molecular Devices Corp., Sunnyvale, CA). 3H uptake was measured using the internal software in a Beckman LSC 6500 counter (Beckman, Brea,CA). Glucose and lactate levels were measured using the internal software in a Bioprofiler Basic (Noval Analytical systems, Niagara Falls, NY). Perturbagens connected to our high-throughput assay results were obtained using CLUE.io (Data version: 1.1.1.2, Software version: 1.1.1.36).
Data analysis	GraphPad Prism (Version 7.03) was used to analyze the data in this study.

For manuscripts utilizing custom algorithms or software that are central to the research but not yet described in published literature, software must be made available to editors/reviewers. We strongly encourage code deposition in a community repository (e.g. GitHub). See the Nature Research guidelines for submitting code & software for further information.

Data

Policy information about availability of data

All manuscripts must include a data availability statement. This statement should provide the following information, where applicable:

- Accession codes, unique identifiers, or web links for publicly available datasets
- A list of figures that have associated raw data
- A description of any restrictions on data availability

The data is available in the Article, Supplementary Information, or from the authors upon reasonable request.

Field-specific reporting

Please select the one below that is the best fit for your research. If you are not sure, read the appropriate sections before making your selection.

- [x] Life sciences
- [ ] Behavioural & social sciences
- [ ] Ecological, evolutionary & environmental sciences

For a reference copy of the document with all sections, see nature.com/documents/nr-reporting-summary-flat.pdf

Life sciences study design

All studies must disclose on these points even when the disclosure is negative.

Sample size	No formal statistical analyses were conducted to determine sample size. Sample sizes were decided upon based on pilot and previous studies.
Data exclusions	No data was excluded from the study.
Replication	All findings were subject to replication, and all replication attempts were successful. All findings could be reproduced.
Randomization	H460 xenograft mice were randomized prior to treatment with Milciclib or vehicle.
Blinding	Most data collection (i.e. cell counts, luminescence measurements) and analyses (i.e. IC50 value determinations) were performed by automated software. The results of data that could not be analyzed by automated software (i.e. 18F-FDG PET images) were confirmed by an individual unaware of the treatment groups.

Reporting for specific materials, systems and methods

We require information from authors about some types of materials, experimental systems and methods used in many studies. Here, indicate whether each material, system or method listed is relevant to your study. If you are not sure if a list item applies to your research, read the appropriate section before selecting a response.

Materials & experimental systems

n/a	Involved in the study	
☐	☒	Antibodies
☐	☒	Eukaryotic cell lines
☒	☐	Palaeontology
☐	☒	Animals and other organisms
☒	☐	Human research participants
☒	☐	Clinical data

Methods

n/a	Involved in the study	
☒	☐	ChIP-seq
☒	☐	Flow cytometry
☒	☐	MRI-based neuroimaging

Antibodies

Antibodies used

HIF-1a: Novus Biologicals; Catalogue number: NB100-105; Clone name: H1alpha67; Lot number: AR-1
GLUT1: Millipore; Catalogue number: 07-1401; Rabbit polyclonal antibody; Lot number: 3085002
GLUT3: Abcam; Catalogue number: ab15311; Rabbit polyclonal antibody
Hexokinase 1: Cell Signaling; Catalogue number: 2024; Clone name: C35C4; Lot number: 3
Hexokinase 2: Cell Signaling; Catalogue number: 2867; Clone name: C64G5; Lot number: 3
CDK2: Cell Signaling; Catalogue number: 2546; Clone name: 78B2; Lot number: 8
CDK4: Cell Signaling; Catalogue number: 12790; Clone name: D9G3E; Lot number: 4
CDK7: Cell Signaling; Catalogue number: 2916; Clone name: MO1; Lot number: 4
TRKA: Cell Signaling; Catalogue number: 2510; Clone name: 12G8; Lot number: 3
PIK3CA: Cell Signaling; Catalogue number: 4249; Clone name: C73F8; Lot number: 10
PTEN: Cell Signaling; Catalogue number: 9559; Clone name: 138G6; Lot number: 19
Phospho-T170 CDK7: Abcam; Catalogue number: ab155976; Clone name: EPR6650(2); Lot number: GR258805-1
Rpb1 NTD: Cell Signaling; Catalogue number: 14958; Clone name: D8L4Y; Lot number: 3
Phospho-S5 Rpb1: Cell Signaling; Catalogue number: 13523; Clone name: D9N5I; Lot number: 1
PKC iota: BD Biosciences; Catalogue number: 610176; Clone name: 23; Lot number: 8162916
Beta Actin: Imgenex; Catalogue number: IMG-5142; Rabbit polyclonal antibody; Lot number: 041418978-02
HA: Abcam; Catalogue number: ab9110; Rabbit polyclonal antibody; Lot number: GR3271390
TXNIP: Cell Signaling; Catalogue number: 14715; Clone name: D5F3E; Lot number: 1

Validation

All primary antibodies were only used for the applications and species (human) recommended by the manufacturer and have been used for the same applications in other publications. Additional evidence of the validity of specific antibodies includes:

- The two antibodies used for ChIP (Rpb1 NTD and phospho-S5 Rpb1) have previously been used by the manufacturer for a ChIP-Seq experiment and show the expected enrichment of signal at the transcriptional start site.
- The HIF-1a, GLUT1, GLUT3, PTEN, and PIK3CA antibodies demonstrate the expected increase in signal intensity in cells treated to induce or over-express HIF-1a, GLUT1, GLUT3, PTEN, and PIK3CA, respectively.
- The CDK2, CDK4, CDK7, TRKA, and PKCi antibodies demonstrate the expected decrease in signal intensity in cells treated with validated CDK2, CDK4, CDK7, TRKA, and PKC iota shRNA constructs, respectively.
- The Hexokinase 1 and 2 antibodies are the same ones that were used in PMID 29880505, where the authors show the expected loss of signal in H460 cells in which Hexokinase 1 or Hexokinase 2 have been knocked out using CRISPR/Cas9 (Figure 5 of that paper).
- The manufacturer's website for the phospho-T170 CDK7 antibody shows loss of signal following the treatment of HeLa cell lysate with lamba phosphatase.
- The HA antibody used for ChIP is ChIP grade.
- The Beta Actin antibody has been used in other publications (i.e. PMID 30463940), and the signal from this antibody was never significantly different between samples in which we loaded the same amount of protein on the gel.
- The TXNIP antibody is the same used in PMID 30197082 where the authors show the expected loss of signal for the antibody in TXNIP knockout cells (Figure 4D of that paper).

Eukaryotic cell lines

Policy information about cell lines

Cell line source(s)	All cell lines were purchased from ATCC.
Authentication	All of the cell lines used were from fresh vials of cells purchased from ATCC. No further authentication was performed.
Mycoplasma contamination	All cell lines tested negative for mycoplasma contamination.
Commonly misidentified lines (See ICLAC register)	None

Animals and other organisms

Policy information about studies involving animals; ARRIVE guidelines recommended for reporting animal research

Laboratory animals	Species: Mus musculus; Strain: NOD scid gamma; Sex: Female; Age: 8 weeks old
Wild animals	The study did not involve wild animals.
Field-collected samples	The study did not involve samples collected from the field.
Ethics oversight	All mouse experiments were approved by the UCLA Animal Research Committee.

Note that full information on the approval of the study protocol must also be provided in the manuscript.
